# Structure of the human activated spliceosome in three conformational states

**DOI:** 10.1038/cr.2018.14

**Published:** 2018-01-23

**Authors:** Xiaofeng Zhang, Chuangye Yan, Xiechao Zhan, Lijia Li, Jianlin Lei, Yigong Shi

**Affiliations:** 1 Beijing Advanced Innovation Center for Structural Biology, Tsinghua-Peking Joint Center for Life Sciences, School of Life Sciences and School of Medicine, Tsinghua University, Beijing 100084, China; 2 Technology Center for Protein Sciences, Ministry of Education Key Laboratory of Protein Sciences, School of Life Sciences, Tsinghua University, Beijing 100084, China; 3 Institute of Biology, Westlake Institute for Advanced Study, Westlake University, Shilongshan Road No. 18, Hangzhou, Zhejiang 310064, China

**Keywords:** spliceosome, cryo-EM structure, activated spliceosome, B^act^ complex, pre-mRNA splicing

## Abstract

During each cycle of pre-mRNA splicing, the pre-catalytic spliceosome (B complex) is converted into the activated spliceosome (B^act^ complex), which has a well-formed active site but cannot proceed to the branching reaction. Here, we present the cryo-EM structure of the human B^act^ complex in three distinct conformational states. The EM map allows atomic modeling of nearly all protein components of the U2 small nuclear ribonucleoprotein (snRNP), including three of the SF3a complex and seven of the SF3b complex. The structure of the human B^act^ complex contains 52 proteins, U2, U5, and U6 small nuclear RNA (snRNA), and a pre-mRNA. Three distinct conformations have been captured, representing the early, mature, and late states of the human B^act^ complex. These complexes differ in the orientation of the Switch loop of Prp8, the splicing factors RNF113A and NY-CO-10, and most components of the NineTeen complex (NTC) and the NTC-related complex. Analysis of these three complexes and comparison with the B and C complexes reveal an ordered flux of components in the B-to-B^act^ and the B^act^-to-B^*^ transitions, which ultimately prime the active site for the branching reaction.

## Introduction

Pre-mRNA splicing is executed by a dynamic ribonucleoprotein complex known as the spliceosome^1^. The first assembled spliceosome is the pre-catalytic B complex, in which the 5-splice site (5′SS) and the branch point sequence (BPS) of the intron are recognized by U6 and U2 small nuclear ribonucleoproteins (snRNPs), respectively. The B complex lacks a functional active site and cannot proceed to the branching reaction. RNP remodeling of the B complex by the RNA-dependent ATPase/helicase Brr2 results in the dissociation of U1 and U4 snRNPs and the recruitment of about 20 protein components, forming the activated spliceosome (B^act^ complex)^1^. These newly recruited proteins mainly constitute three classes: the NineTeen complex (NTC), the NTC-related (NTR), and the splicing factors^2,3,4^. Despite a well-formed active site, the B^act^ complex still cannot catalyze the branching reaction due to spatial separation of the BPS away from the 5′SS^5,6^. Conversion of the B^act^ complex into the catalytically activated B^*^ complex by the ATPase/helicase Prp2 allows the branching reaction to occur, generating a 5′-exon and an intron lariat-3′-exon intermediate. The resulting catalytic step I spliceosome (C complex) is converted by Prp16 into the step II activated spliceosome (C^*^ complex), which catalyzes the ligation of the 5′-exon with the 3′-exon.

Major mechanistic advances through structural biology have been achieved in the understanding of pre-mRNA splicing in the past 2 years^7,8^. The first structure of an intact spliceosome at a near-atomic resolution — that of the *Schizosaccharomyces pombe* (*S. pombe*) ILS complex at 3.6 Å^9,10^ — reveals a conserved overall organization of the spliceosome and a conserved spatial arrangement of the splicing active site^11^. Subsequent cryo-EM structures of the *Saccharomyces cerevisiae* (*S. cerevisiae*) and *S. pombe* spliceosomes at different stages of the splicing cycle provide important mechanistic information^5,6,12,13,14,15,16,17,18,19,20^. In contrast to the yeast spliceosome, structural information on the human spliceosome has been slow to emerge, in part due to its considerably more dynamic nature. At present, we only have the structures of the human B complex at 9.9 Å^21^ and the human C^*^ complex at 5.9 Å^22^ and 3.8 Å^23^.

In this manuscript, we report the cryo-EM structures of the human B^act^ complex in three distinct compositional and conformational states at resolutions of 4.9, 5.1, and 6.5 Å. These structures allow mechanistic understanding of the dynamic steps surrounding formation of the human B^act^ complex and its transitions from the B complex and to the B^*^ complex.

## Results

### Spliceosome isolation and electron microscopy

An *in vitro* splicing assay was employed to assemble the human spliceosomes on an intact, synthetic pre-mRNA. The spliceosomal sample (named sample I hereafter) were found to contain a mixture of the human B^act^, C, and C^*^ complexes^24^. The cryo-EM structure of the human C complex was determined from this sample^24^. The RNP remodeling from the B to the B^act^ complex involves flux of several dozen protein and RNA components and is thought to occur in distinct steps^20,21^. To gain insights into this dynamic process, we deleted the 3′-exon from the synthetic pre-mRNA with only 19 nucleotides downstream of the BPS such that Prp2 is unable to grab the RNA sequences for the B^act^-to-B^*^ conversion^25^. Using the truncated pre-mRNA, we prepared a second batch of the cryo-EM sample (sample II, hereafter). Unlike sample I, sample II is predicted not to contain any spliceosomes beyond the B^act^ complex and thus may yield information on the assembly of the human B^act^ complex. Sample II was purified by affinity chromatography followed by glycerol gradient centrifugation (Supplementary information, Figure S1A). To maintain the structural integrity of the spliceosomes, chemical crosslinking by glutaraldehyde was applied to the sample during centrifugation. After removal of the glycerol, sample II was examined by negative staining EM (Supplementary information, Figure S1B) and used for cryo-EM sample preparation. Micrographs were collected using the K2 Summit detector mounted on a Titan Krios microscope (Supplementary information, Figure S1C).

We first processed the data set derived from sample I, which has 1 464 033 particles^24^ (Supplementary information, Figure S2). Following three parallel runs of multi-reference three-dimensional (3D) classification and subsequent local 3D classifications, 49 218 particles yielded a reconstruction of the human B^act^ complex at an average resolution of 4.8 Å. A follow-up 3D classification with a soft mask in the RNF113A region led to the identification of two major conformational states at average resolutions of 5.1 Å and 6.5 Å on the basis of the FSC value 0.143 (Supplementary information, Figures S2 and S4; Tables S1 and S2). As will be detailed later, the 5.1-Å and 6.5-Å reconstructions represent the mature and late human B^act^ complexes, respectively.

Next, we processed the data set from sample II, which yielded 629 472 particles (Supplementary information, Figure S3). As anticipated, only one dominant spliceosomal complex — the B^act^ complex — is present. Using a similar data processing strategy, 96 523 particles yielded a reconstruction of the early B^act^ complex at an average resolution of 4.9 Å (Supplementary information, Figures S3 and S4; Tables S1 and S2). Despite apparent differences among the three conformational states of the human B^act^ complex (Supplementary information, Figure S5), they share the same structure in the SF3b region. Combining both data sets, we improved the local resolution to 4.2 Å in the SF3b region (Supplementary information, Figures S3, S4A and S6). Atomic modeling of the human B^act^ complexes was facilitated by the local resolutions of about 4.0-4.5 Å at the core and the SF3b region of the B^act^ complex (Supplementary information, Figures S4B and S6). In addition, the atomic coordinates of the yeast B^act^^5^ and the human C^*^ complex^23^ greatly expedited modeling of the human B^act^ complexes.

### Overall structure

**Figure 1:**
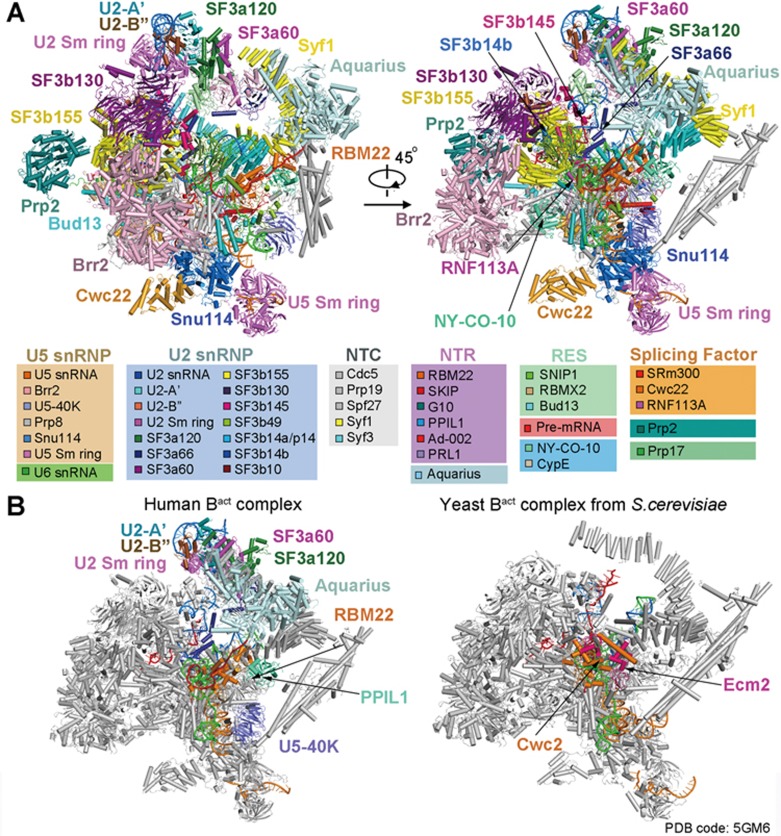
Cryo-EM structure of the human activated spliceosome (the B^act^ complex). **(A)** Two views of the human mature B^act^ complex. The protein and RNA components are color-coded and tabulated below the images. The structure of the mature B^act^ complex shown here includes 52 proteins, three snRNAs, and one pre-mRNA, with a combined molecular mass of about 1.8 MDa. U2, U5, and U6 snRNAs are colored marine, orange, and green, respectively. Pre-mRNA is colored red. This coloring scheme is preserved throughout this manuscript. **(B)** Structural comparison between the human and yeast B^act^ complexes^5,6^. For protein components, only those that are unique in either spliceosome are colored. All shared protein components are shown grey. All structural images were created using PyMol^49^.

The final model of the human mature B^act^ complex contains 15 479 amino acids from 52 proteins and 414 nucleotides from three snRNAs and the pre-mRNA (Figure 1A; Supplementary information, Tables S1 and S2), with a combined molecular weight of about 1.8 mega-Daltons. The 52 protein components include all 11 from U5 snRNP, 19 from U2 snRNP, five from the NTC, seven from the NTR, three from the retention and splicing (RES) complex (SNIP1, Bud13, and RBMX2), three splicing factors (SRm300, Cwc22, and RNF113A), two peptidyl prolyl isomerases (PPIs, NY-CO-10, and CypE), the ATPase/helicase Prp2, and the step II factor Prp17. The U2 snRNP includes all seven proteins of the SF3b complex (SF3b155, SF3b145, SF3b130, SF3b49, SF3b14a/p14, SF3b14b, and SF3b10), three proteins of the SF3a complex (SF3a120, SF3a66, and SF3a60), U2 snRNA, and nine proteins of the U2 snRNP core that only interact with U2 snRNA (U2-A′, U2-B′′, and the heptameric U2 Sm complex) (Figure 1A).

The overall appearance of the human mature B^act^ complex closely resembles that of the *S. cerevisiae* B^act^ complex^5,6^ (Figure 1B). Compared to the *S. cerevisiae* complex^5^, the structure of the human mature B^act^ complex contains 16 additional protein components: SF3a120 and SF3a60 of the SF3a complex, SF3b14a/p14 of the SF3b complex, nine proteins of the U2 snRNP core, U5-40K of the U5 snRNP, and three proteins of the NTR (Aquarius, RBM22, and PPIL1) (Figure 1B). Of these proteins, RBM22 appears to have arisen from a fusion event between the two yeast NTR proteins Cwc2 and Ecm2^26^. The N-terminal zinc-binding domain and the C-terminal RRM domain of RBM22 share significant sequence homology with Ecm2 and Cwc2, respectively.

**Figure 2:**
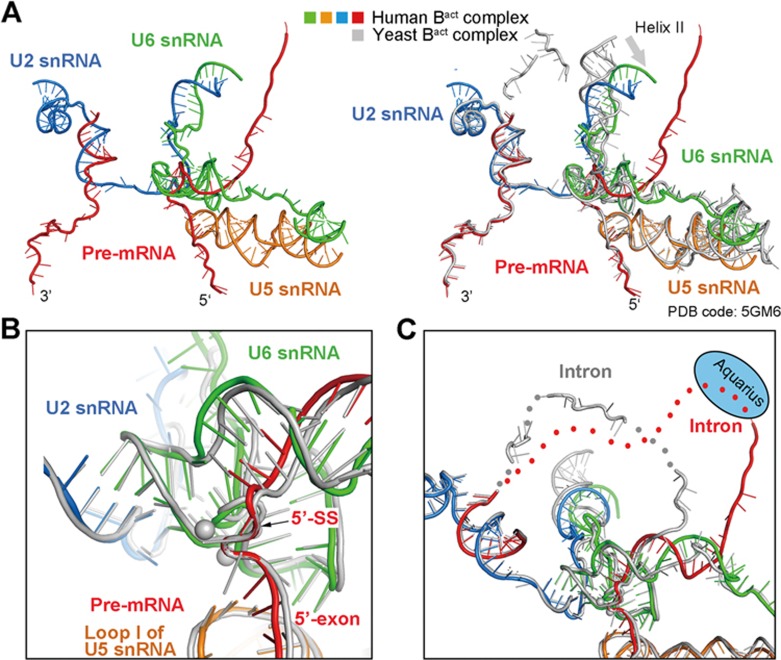
The RNA elements and the splicing active site of the human mature B^act^ complex. **(A)** Structure of the RNA elements in the core of the human mature B^act^ complex. The color-coded RNA elements of the human B^act^ complex are shown in the left panel, and their superposition with those of the *S. cerevisiae* B^act^ complex^5^ is displayed in the right panel. All yeast RNA elements are colored grey. The helix II of the U2/U6 duplex in the human B^act^ complex is bent relative to that in the yeast complex. **(B)** Structural overlay of the active site RNA elements between the human and *S. cerevisiae* B^act^ complexes^5^. **(C)** The U6/intron duplex in the human B^act^ complex is considerably longer than that in the *S. cerevisiae* B^act^ complex^5^.

The RNA elements in the human mature B^act^ complex adopt a generally similar conformation as that in the yeast B^act^ complex^5,6^ (Figure 2A). The similarity extends to the fine local conformations of the active site RNA elements (Figure 2B). One notable difference is the helix II of the U2/U6 duplex, which is bent by about 40° in the human B^act^ complex relative to that in yeast (Figure 2A). Another marked difference is an extra turn of the U6/intron duplex in the human B^act^ complex beyond the U6/5′SS duplex, which results in the separation of the downstream human intron sequences away from those in *S. cerevisiae* by up to 40 Å (Figure 2C). In addition, similar to the human C^*^ complex^23^, the intron sequences are locked by RBM22 through a positively charged central cavity in the mature and late B^act^ complexes, but not in the early B^act^ complex (Supplementary information, Figure S7). Because the loading of RBM22 occurs in the transition from the early to mature B^act^ complex, RBM22 must undergo partial unfolding to enclose the intron sequences that are already bound in the early B^act^ complex.

### Three conformational states of the human B^act^ complex

**Figure 3:**
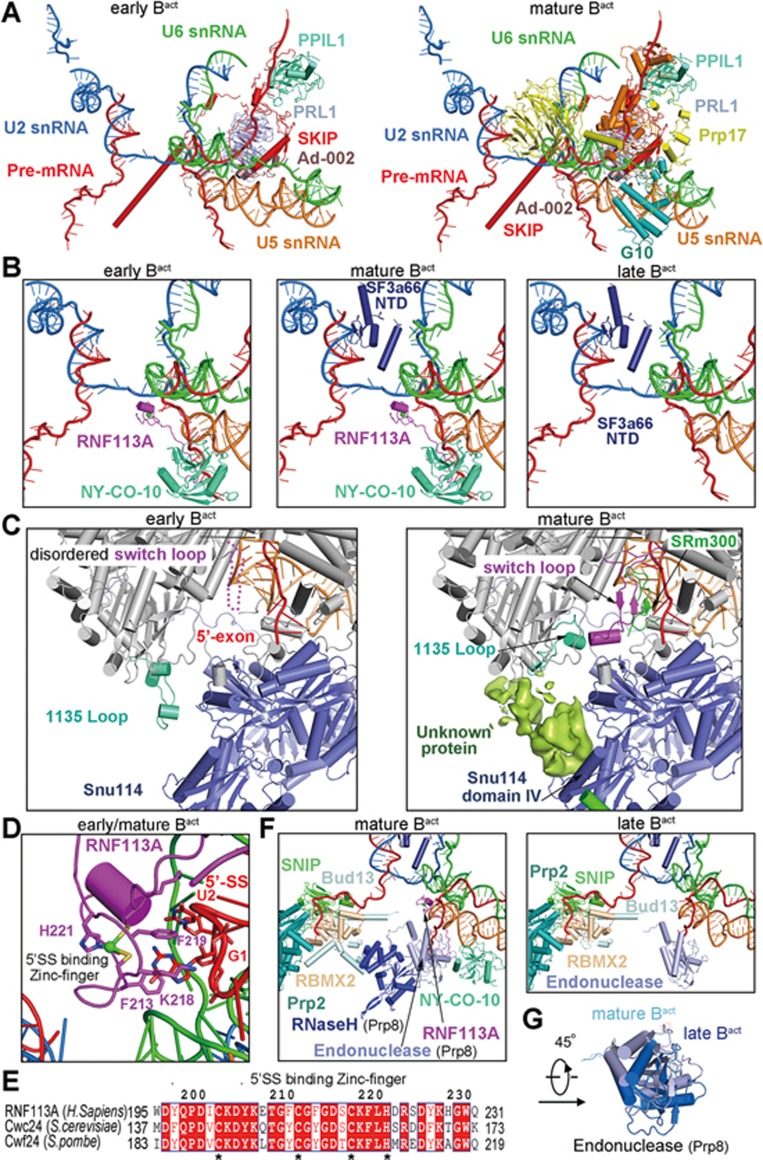
The early, mature, and late B^act^ complexes represent three different conformational states of the human spliceosome. **(A)** Structural comparison between the early B^act^ complex (left panel) and the mature B^act^ complex (right panel). Shown here are only the RNA elements and the protein components that undergo dynamic changes in the core of the spliceosome in the transition of the early to mature B^act^ complex. Compared to that of the early B^act^ complex, the core of the mature B^act^ complex contains four additional proteins: G10, Prp17, RBM22, and the N-terminal domain (NTD) of the SF3a component SF3a66. **(B)** Close-up views on the early (left panel), mature (middle panel), and late (right panel) B^act^ complexes. Notably, the splicing factors RNF113A (Cwc24 in *S. cerevisiae*) and NY-CO-10 (Cwc27 in *S. cerevisiae*) are loaded in the early and the mature, but not the late, B^act^ complexes. On the other hand, SF3s66 is loaded in the mature and late B^act^ complexes, but not the early B^act^ complex. **(C)** A close-up comparison of the Switch loop regions between the early and mature B^act^ complexes. The Switch loop of Prp8 is stabilized by an extended sequence (named 1135-loop) and the splicing factor SRm300 (Cwc21 in *S. cerevisiae*) in the mature B^act^ complex (right panel). SRm300 is absent and the 1135-loop is shifted away in the early B^act^ complex (left panel); consequently the Switch loop is flexible and remains unidentified. **(D)** A close-up view on the recognition of the guanine nucleotide (G1) at the 5-end of the 5′-splice site (5′SS) by the splicing factor RNF113A. The stacking of the guanine base against the aromatic rings of Phe213 and Phe219 of RNF113A is reminiscent of that in the yeast B^act^ complex^5^. **(E)** Sequence alignment between the human RNF113A and its yeast orthologues Cwc24 (*S. cerevisiae*) and Cwf24 (*S. pombe*). The three key residues involved in recognition of G1 of the 5′SS (Phe213, Lys218, and Phe219) are highly conserved. **(F)** Close-up views on the role of the endonuclease-like domain and the RNaseH-like domain of Prp8 in the mature and late B^act^ complexes. In the mature B^act^ complex (left panel), both domains of Prp8 appear to stabilize the binding of RNF113A and NY-CO-10 in the spliceosome. The RNaseH-like domain also binds Bud13 of the RES complex. In the late B^act^ complex (right panel), RNF113A and NY-CO-10 have been dissociated, leading to the dislocation of the RNaseH-like domain and Bud13. **(G)** Superposition of the endonuclease-like domain of Prp8 between the mature and late B^act^ complexes. The core machineries of the two complexes are aligned.

Despite nearly identical conformation of the snRNA elements and the pre-mRNA, the early, mature, and late B^act^ complexes can be conclusively differentiated on the basis of their protein components (Figure 3A). First, the splicing factor Prp17 and the NTR proteins RBM22 and G10 (Bud31 in yeast) are fully loaded in the mature and late B^act^ complexes, but not in the early B^act^ complex (Figure 3A; Supplementary information, Figure S7). These three proteins — G10, Prp17, and RBM22 — help stabilize the active site RNA elements and are present in the human C and C^*^ complexes^23,24^. Their absence strongly suggests the premature nature of the early B^act^ complex. Second, the N-terminal domain (NTD) of the SF3a component SF3a66 (Prp11 in yeast) is present in the mature and late B^act^ complexes, but not in the early B^act^ complex (Figure 3B; Supplementary information, Figure S8). In addition, four proteins of the NTC (Prp19, Syf1, Spf27, and Cdc5), two components of the NTR (PPIL1 and Aquarius), and the PPI protein CypE are fully loaded in the mature and late B^act^ complexes, but not in the early B^act^ complex (Supplementary information, Figure S9). Perhaps most importantly, the Switch loop in Prp8 of the mature and late B^act^ complexes is positioned identically as that in the C or C^*^ complex^23,24^ and interacts with the splicing factor SRm300 (Cwc21 in yeast) (Figure 3C). In contrast, SRm300 is yet to be loaded into the early B^act^ complex and the Switch loop remains flexible with no obvious EM density. Collectively, these structural differences unequivocally identify the premature nature of the early B^act^ complex.

Compared to the mature B^act^ complex, the late B^act^ complex no longer contains the splicing factors RNF113A (Cwc24 in yeast) and NY-CO-10 (Cwc27 in yeast) (Figure 3B; Supplementary information, Figure S10). This structural finding is consistent with the biochemical observation that these two proteins only transiently associate with the spliceosome and are released during the B^act^-to-B^*^ transition^27^. Intriguingly, these two splicing factors are strongly present in the early B^act^ complex, suggesting a role of RNF113A in organizing the active site during spliceosome activation^28^. Careful examination reveals that, compared to the early B^act^ complex, the EM density for these two splicing factors is already weakened in the mature B^act^ complex (Supplementary information, Figure S10B and S10C). This analysis further suggests that, during the B^act^-to-B^*^ transition, the splicing factors RNF113A and NY-CO-10 are likely released ahead of all other components. In the early and mature B^act^ complexes, the guanine base of G1 stacks closely against the aromatic side chains of Phe213 and Phe219 from RNF113A (Figure 3D). These two aromatic residues, together with Lys218 that also stabilizes the 5′SS, come from the zinc-binding domain of RNF113A and are invariant in the yeast orthologues (Figure 3E). Because RNF113A directly protects the 5′-end guanine base of the 5′SS, its release may signal the beginning phase of the B^act^-to-B^*^ transition. Therefore, the late B^act^ complex likely represents the state of the spliceosome just preceding its transition to the B^*^ complex through the action of Prp2. Because the early B^act^ complex was obtained using a shortened pre-mRNA, Prp2 may be required for the conversion of the early to the mature and late B^act^ complexes. Intriguingly, the N-terminus of RNF113A is buried in the cleft between the endonuclease-like domain and the N-domain of Prp8 (Supplementary information, Figure S10A and S10B); therefore the release of RNF113A requires conformational changes in Prp8, which is confirmed in the late B^act^ complex.

The compositional changes in the core of the spliceosome also cause pronounced conformational and positional shifts for the surrounding protein components. For example, in the mature B^act^ complex, the RNaseH-like domain of Prp8 interacts with the endonuclease-like domain, which directly binds to NY-CO-10 (Figure 3F). The RNaseH-like domain also associates with Bud13 of the RES complex. With the dissociation of RNF113A and NY-CO-10 in the late B^act^ complex, the RNaseH-like domain and Bud13 have been dislocated (Figure 3F), and the endonuclease-like domain of Prp8 also undergoes a 45° rotation (Figure 3G).

### The SF3a and SF3b complexes

**Figure 4:**
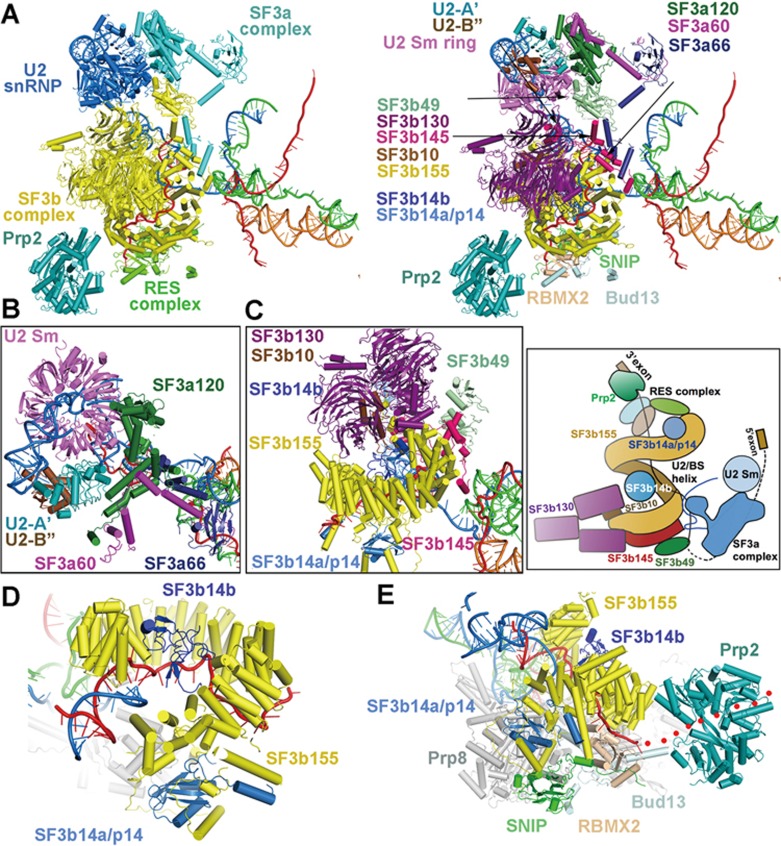
Structures of the SF3a and SF3b complexes. **(A)** Structure of the SF3a and SF3b complexes in the context of key surrounding components. In the left panel, the SF3a and SF3b complexes are colored cyan and yellow, respectively. The U2 snRNA, the RES complex, and Prp2 are colored blue, green, and teal, respectively. The RNA elements are displayed for orientation. In the right panel, the individual components of the SF3a and SF3b complexes are color-coded and labeled. The SF3a complex consists of three proteins: SF3a60, SF3a66, and SF3a120. The SF3b complex comprises seven proteins SF3b10, SF3b14a/p14, SF3b14b, SF3b49, SF3b130, SF3b145, and SF3b155. **(B)** A close-up view on the SF3a complex and its interactions with the U2 snRNP subcomplex involving U2 Sm ring. **(C)** A close-up view on the SF3b complex and the interactions among its constituents. The structure is shown in the left panel and the cartoon representation is displayed in the right panel. **(D)** A close-up view on the components SF3b14b and SF3b14a/p14 of the SF3b complex. **(E)** A close-up view on the 3′-end sequences of the pre-mRNA and nearby protein components. The 3′-end sequences of the pre-mRNA are bound by RBMX2 of the RES complex. The dotted lines leading to the RNA-binding groove of Prp2 indicate the path of the RNA sequences downstream of the last ordered nucleotide in the structure.

The SF3a and SF3b complexes are major constituents of the U2 snRNP. The SF3a complex plays an important role in the formation of the splicing active site in the B^act^ complex and interacts with the SF3b complex and the U2 snRNP core (Supplementary information, Figure S11). Only one component of the SF3a complex — Prp11 in *S. cerevisiae* (SF3a66 in human) — was structurally resolved in the yeast spliceosome^5^. In the structure of the human B^act^ complex, all three components — SF3a120, SF3a66, and SF3a60 — are unambiguously identified (Figure 4A). All three proteins exhibit extended conformations, with SF3a60 bridging the gap between SF3a120 and SF3a66. Three α-helices at the N-terminal half of SF3a60 closely interact with the α-helices at one end of SF3a120, stabilizing its extended conformation. Subsequently, an α-helix of SF3a60 directly contacts the β-sandwich domain of SF3a66, with the ensuing extended sequences of SF3a60 wrapping around the β-sandwich (Figure 4A and 4B). Notably, SF3a66 is the only SF3a protein that specifically recognizes an RNA element — the U2/intron duplex. SF3a120 adopts an all-α-helical conformation and associates with U2-A′ (Lea1 in yeast) and the U2 heptameric Sm complex. Similar to that in the *S. cerevisiae* B^act^ complex^5^, the N-terminus of SF3a66 reaches into the active site and directly contributes to the coordination of the G1 nucleotide of the 5′SS.

The SF3b complex directly recognizes the BPS and surrounding intron sequences^29,30,31^. In the human B^act^ complex, all seven components of the SF3b are structurally resolved, including SF3b155 (Hsh155 in *S. cerevisiae*), SF3b145 (Cus1 in *S. cerevisiae*), SF3b130 (Rse1 in *S. cerevisiae*), SF3b49 (Hsh49 in *S. cerevisiae*), SF3b14a/p14, SF3b14b (Rds3 in *S. cerevisiae*), and SF3b10 (Ysf3 in *S. cerevisiae*)^32^. These seven proteins assemble into a compact subcomplex (Figure 4C). Importantly, SF3b14a/p14 is unique to the human spliceosome and absent in *S. cerevisiae* B^act^ complex^33^. In contrast to previous assignment^34,35^, SF3b14a/p14 is located at the periphery, not the center, of the SF3b complex in the human B^act^ complex and is surrounded by three extended N-terminal helices and N-terminal HEAT repeats of SF3b155 (Figure 4C and 4D).

Other than SF3b14a/p14, the other components of the human SF3b complex are located in generally the same positions as those of the *S. cerevisiae* SF3b complex^5,6,34^ (Figure 4C). Similar to its *S. cerevisiae* orthologue Hsh155, SF3b155 contains an N-terminal helix-loop-helix (N-HLH) and 20 HEAT repeats (Supplementary information, Figure S12A). The N-HLH domain is sandwiched between the RT Finger/Palm and the Linker domains of Prp8, and interacts with SKIP and components of the RES complex (Supplementary information, Figure S12B). Compared to Hsh155, SF3b155 contains two extra sequence elements: a Trp-rich motif and a p14-binding motif (Supplementary information, Figure S12A). The extended p14-binding sequences also interact with SKIP, SNIP of the RES complex, and the RT Finger/Palm domain of Prp8 (Supplementary information, Figure S12C). The HEAT repeats, each comprising a pair of anti-parallel α-helices, constitute a left-handed superhelical structure and serves as the central scaffold of the SF3b complex by interacting with a number of protein and RNA components (Supplementary information, Figure S12D).

SF3b10 binds the C-terminal α-helices of SF3b155, whereas SF3b145 in an extended conformation stabilizes six HEAT repeats at the C-terminus of SF3b155 on the outside of the superhelical structure (Figure 4C). SF3b49 binds SF3b145 from the outside and interacts with the upstream sequences of the BPS, stabilizing the U2/BPS duplex (Figure 4C and 4D). The N-terminal and C-terminal WD40 domains of the Y-shaped SF3b130 sandwich SF3b10 and the C-terminal α-helix of SF3b155. This structural arrangement places the C-terminal WD40 domain of SF3b130 in direct contact with one end of SF3b145. SF3b14b is bound in the hollow center of the SF3b155 superhelical structure and directly interacts with the N-terminal WD40 domain of SF3b130.

The U2/BPS duplex is bound to SF3b155 through a lateral opening of its superhelical structure (Figure 4D). The RNA sequences downstream of the BPS traverse through the hollow center of the SF3b155 spiral, contacting residues from both SF3b14b and SF3b155, and come out of the other side of the spiral. The following intron sequences skim over the surface of RBMX2 (Snu17 in *S. cerevisiae*) of the RES complex (Figure 4E). The human RES complex, with a critical role in the splicing and retention of pre-mRNA^36,37^, consists of SNIP, RBMX2, and Bud13^38^ and closely interacts with the SF3b complex. The RNA sequences downstream of those bound by RBMX2 would presumably reach the RNA-binding groove of the ATPase/helicase Prp2 (Figure 4E).

### The B-B^act^-C transition

**Figure 5:**
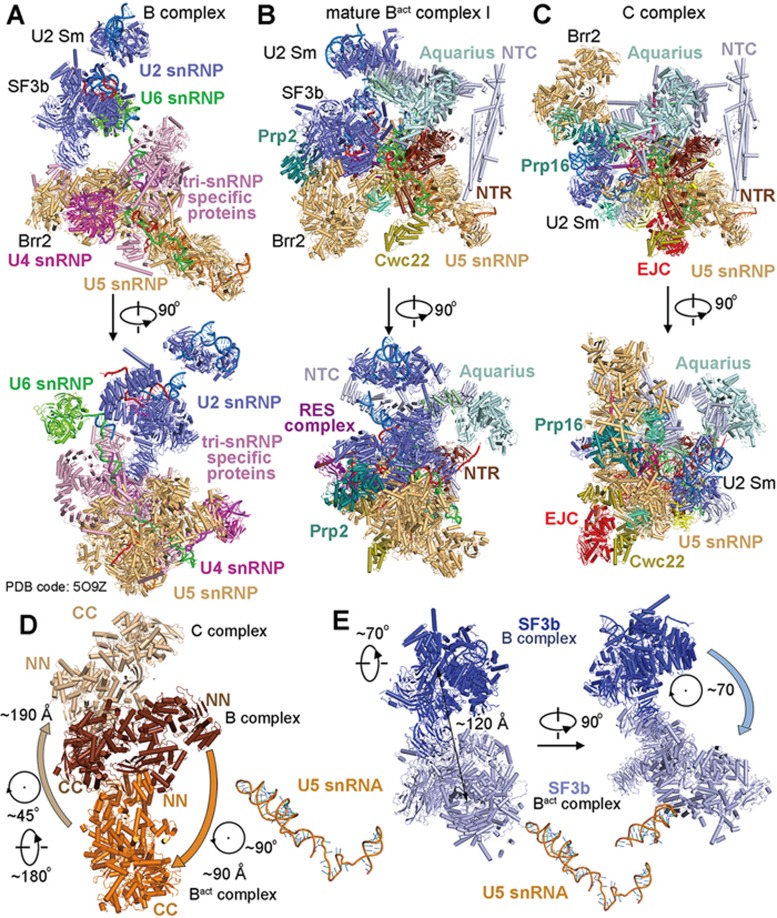
Structural comparison among the B complex, the B^act^ complex, and the C complex. **(A)** Structure of the human B complex^21^. Two perpendicular views are shown. The tri-snRNP-specific proteins, and U2, U4, U5, and U6 snRNPs are colored pink, blue, orange, magenta, and green, respectively. **(B)** Structure of the human mature B^act^ complex. Two perpendicular views are shown, and these two views are identical to those in **C** of the human C complex^24^. Ribonucleoprotein remodeling from the B to the B^act^ complex is the most dramatic in the splicing cycle, involving dissociation of the U4 snRNP and tri-snRNP-specific proteins and recruitment of the NTC and NTR components along with several splicing factors and the ATPase/helicase Prp2. **(C)** Structure of the human C complex^24^. Compared to the B^act^ complex, the SF3a/SF3b complexes along with Prp2 and the splicing factor RNF113A have been dissociated, and the exon junction complex (EJC) along with the step I factors CCDC49/CCDC94 and the ATPase/helicase Prp16 have been recruited. **(D)** Movement of the ATPase/helicase Brr2 in the B-B^act^-C transition. The U5 snRNA molecules from the three human spliceosomal complexes are superimposed. **(E)** Movement of the SF3b complex in the B-to-B^act^ transition. The U5 snRNA molecules from the human B and B^act^ complexes are superimposed.

The spliceosomal B-to-B^act^ transition, driven by the ATPase/helicase Brr2^39,40^, is particularly dramatic, involving dissociation of the tri-snRNP-specific proteins and the entire U4 snRNP and recruitment of the NTC and NTR proteins (Figure 5A and 5B). Consequently, the overall appearance of the human B^act^ complex bears little resemblance to that of the human B complex^20,21^. The B^act^-to-B^*^ transition, propelled by the ATPase/helicase Prp2^41^, is less dramatic compared to the B-to-B^act^ transition but involves flux of considerably more proteins than the C-to-C^*^ and P-to-ILS transitions, which is driven by the ATPase/helicases Prp16 and Prp22, respectively^42,43,44^. Virtually all components of the SF3a and SF3b complexes, along with Prp2 and the splicing factors RNF113A and NY-CO-10, are dissociated in the B^act^-to-B^*^ transition. Prp16 and the step I factors CCDC49 and CCDC94 are recruited into the B^*^ complex. At present, the B^*^ complex remains the only structurally uncharacterized spliceosome during the splicing cycle. Fortunately, the structure of the B^*^ complex is predicted to be nearly identical to that of the C complex except in the active site region surrounding the 5′SS and the BPS where the branching reaction occurs^12^. There is no change of protein components between the B^*^ and C complexes. Therefore, structural comparison between the B^act^ and C complexes (Figure 5B and 5C) should recapitulate many of the essential features of the B^act^-to-B^*^ transition. Despite the flux of more than one dozen proteins, the overall appearance of the human B^act^ complex is similar to that of the C complex^24^, particular in the core region and on the side of the NTC core and U5 snRNP.

Due to the dramatic remodeling, components of the spliceosome have undergone major positional adjustment. Brr2, e.g., is rotated 90° and translocated by about 90 Å in the B-to-B^act^ transition, and is swirled and shifted by approximately 190 Å in the B^act^-to-C transition (Figure 5D). The entire SF3b complex undergoes a 70° rotation followed by a 120-Å translocation in the B-to-B^act^ transition (Figure 5E). Intriguingly, the sequences near the 3′-end of U2 snRNA form two short stems loop structures known as IIa and IIb in the B complex and remain unchanged in the B^act^ complex; however, these sequences constitute a long stem loop known as IIc in the C complex (Supplementary information, Figure S13). This structural finding is consistent with the biochemical observation that U2 IIa promotes spliceosome assembly whereas U2 IIc facilitates the branching reaction^45,46,47^.

## Discussion

In *S. cerevisiae*, only about 4% of the protein-encoding genes contain introns^48^. In contrast, most of the protein-encoding genes in the human genome contain introns. Pre-mRNA splicing in human is considerably more complex than that in yeast and is subject to more stringent regulation. Accordingly, the human spliceosome is compositionally and conformationally more dynamic compared to the yeast spliceosome. In this study, using synthetic pre-mRNA in the absence or presence of the 3′-exon, we were able to obtain two samples for cryo-EM analysis. The sample prepared using the intact pre-mRNA gave rise to the B^act^, C, and C^*^ complexes^24^, of which the B^act^ complex represent the mature and late states. In contrast, the sample prepared using the 3′-exon-deleted pre-mRNA only yielded one dominant spliceosome species — the early B^act^ complex.

The definition for these three conformational states of the B^act^ complex is justified not only by the method of spliceosome assembly but also by the actual compositions of the spliceosome (Figure 3). Importantly, the Switch loop of Prp8 is positioned similarly as that of the human C complex^24^ only in the mature/late, but not the early, B^act^ complex. The splicing factor SRm300, which stabilizes the Switch loop, is loaded similarly as that of the human C complex^24^ only in the mature/late, but not the early, B^act^ complex. Another structural observation is the presence of RBM22 in the mature/late, but not the early, B^act^ complex. Consequently, the intron sequences are only interlocked by RBM22 in the mature/late, but not the early, B^act^ complex (Supplementary information, Figure S7). These mutually coherent structural observations are fully consistent with the reaction coordinate of the spliceosome and the requirement of the splicing reaction.

**Figure 6:**
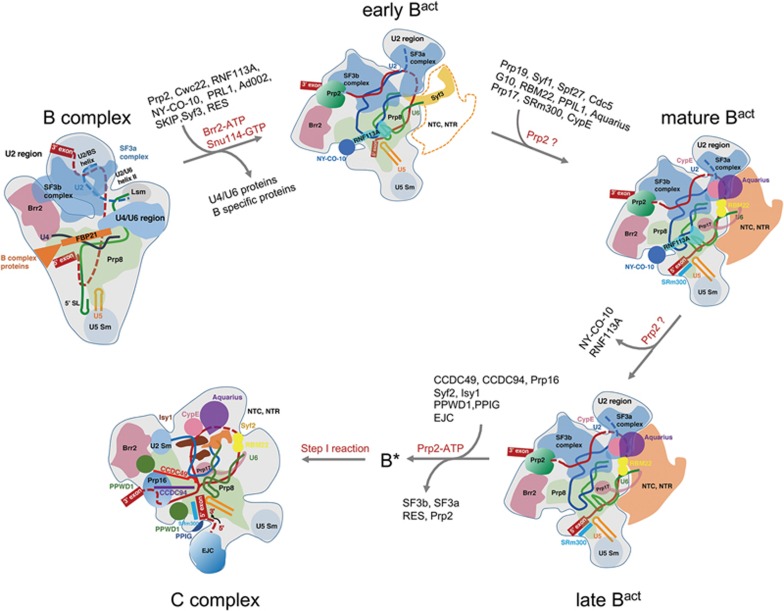
A structure-based model of the ribonucleoprotein remodeling from the B complex to the C complex. The B-to-B^act^ transition represents the most complex transition in the pre-mRNA splicing cycle^1^. The ATPase/helicase Brr2 drives the formation of the early B^act^ complex, where the NTC and NTR components are yet to be recruited. In the active site of the early B^act^ complex, the splicing factor RNF113A and the PPI NY-CO-10 are already loaded but the N-terminal domain (NTD) of SF3a66 along with G10 and Prp17 are yet to be recruited. Next, components of the NTC and NTR, Prp17, along with the NTD of SF3a66, are recruited to form the mature B^act^ complex.

Analysis of the three conformational states of the B^act^ complex suggests an ordered transition from the pre-catalytic B complex to the B^*^ complex (represented by the C complex) (Figure 6). In the first step, driven by the ATPase/helicase Brr2, the tri-snRNP-specific components, the U4 snRNP, and proteins of the U6 snRNP are dissociated from the B complex. About 10 proteins — Ad-002, Cwc22, NY-CO-10, PRL1, Prp2, RNF113A, SKIP, Syf3, and the RES complex — are recruited, forming the early B^act^ complex (Figure 6). Remarkably, the majority of the NTC and NTR proteins remain unbound in the early B^act^ complex. Formation of the early B^act^ complex is presumably transient because it is absent in the cryo-EM sample that was prepared using the intact pre-mRNA. Despite its transient nature, the early B^act^ complex was trapped through the use of a 3′-exon-deleted pre-mRNA. The inability for Prp2 to bind and pull the 3′-end of the pre-mRNA likely allows the accumulation of this otherwise transient B^act^ species. This analysis further suggests that the ensuing steps after the early B^act^ complex may require the action of Prp2.

In the second step, the remaining NTC and NTR proteins, together with the NTD of SF3a66, the splicing factors SRm300 and Prp17 and the PPI CypE, are recruited, forming the mature B^act^ complex (Figure 6). In the third step, the splicing factors RNF113A and NY-CO-10 are released, leading to the late B^act^ complex. As suggested earlier, the second and third steps may both require the binding of the pre-mRNA by Prp2. We speculate that the flux of protein components in both steps may be greatly facilitated by the ATP hydrolysis-propelled pulling, which likely allows the empty binding sites to be more accessible to the incoming proteins. In the fourth and last step, through the action of Prp2, the SF3a complex, the SF3b complex, and the RES complex are dissociated, leading to the release of Prp2. The vacated space likely allows the recruitment of the step I-specific factors CCDC49 and CCDC94, the NTC proteins Sfy2 and Isy1, the exon junction complex, and the PPIs PPWD1 and PPIG (Figure 6).

In summary, structural determination of three conformational and compositional states of the human B^act^ complex facilitates mechanistic understanding of the transitions from the B to B^act^ complex and from the B^act^ to B^*^ complex. Compared to the *S. cerevisiae* B^act^ complex, the 16 additional protein components in the human complex allows meaningful comparison and derivation of conclusions that are unique to higher eukaryotes. Such differences may empower future efforts that are designed to modulate the function of the spliceosome in potential therapeutic intervention of human genetic diseases.

## Materials and Methods

### *In vitro* splicing reaction

*In vitro* splicing with a shortened 3′-tail of the intron in the synthetic pre-mRNA allows assembly of the B^act^ complex but not its catalytic activation^4,25,49^. To capture the B^act^ complex, the 3′-exon in the pre-mRNA MINX-GG^23^ was deleted to generate the MINX-15 pre-mRNA construct. The MS2-binding sites were positioned 46 nucleotides downstream of the 5′-splice site (5′SS) and 52 nucleotides upstream of the BPS as previously described^23^. The M7G(5′)ppp(5′)G-capped pre-mRNA was synthesized in the T7 runoff transcription using a template generated from the PCR reaction; the DNA template was then digested by RNase-free DNase I (Promega) while the RNA was further purified by PCI-extraction and ethanol precipitation. Splicing-active nuclear extract was prepared from HeLa S3 cells as described^50^. *In vitro* splicing reaction was performed in the presence of 15 nM MINX-15 pre-mRNA and 40% nuclear extract in a buffer that contains 20 mM HEPES-KOH, pH 7.9, 2 mM ATP, 20 mM creatine phosphate, 70 mM KCl, 3.5 mM MgCl_2_, and was incubated at 30 °C for 2 h.

### Purification and crosslinking of the spliceosomal complexes

After spliceosome formation, the free pre-mRNA that had not been incorporated into the spliceosome was digested by endogenous RNase H with the addition of two DNA oligonucleotides (MINX cmd1 & cmd2) that are complementary to the upstream sequence of the 5′SS. The resulting solution was quenched on ice and incubated with the amylose resin (NEB) for 2 h. After extensive washing with the HS150 buffer (20 mM HEPES-KOH, pH 7.9, 150 mM NaCl, 1.5 mM MgCl_2_, 4% glycerol), the spliceosome was eluted using 20 mM maltose.

For cryo-electron microscopy (cryo-EM) study, the eluted spliceosomal complexes were loaded onto a 38.6-mL 10%-30% linear glycerol gradient in the G150 buffer (20 mM HEPES-KOH, 150 mM NaCl, 1.5 mM MgCl_2_) supplemented with 0%-0.1% EM-grade glutaraldehyde^51^. Crosslinking, in our case by glutaraldehyde, is essential for maintenance of the human spliceosome integrity. After centrifugation at 4 °C for 13 h at 25 300 rpm in a SW32 rotor (Beckman Coulter), the sample was manually fractionated from top to bottom. The total RNA in each fraction was extracted and analyzed on an 8% denaturing polyacrylamide gel (Supplementary information, Figure S1A). Fractions containing the B^act^ complex were pooled and concentrated using a 100-kDa cut-off centrifugation filter unit (Amicon Ultra) to a volume of 500 μL. Glycerol was removed by dialysis of the sample against the G150 buffer using a 10-kDa Mini-lyzer (Pierce) for at least 5 h.

### EM sample preparation and data acquisition

After removal of glycerol, the spliceosomal complexes were further concentrated to about 0.12 mg/mL for EM sample preparation. Uranyl acetate (2% w/v) was used for negative staining. Briefly, the copper grids supported by a thin layer of carbon film (Zhongjingkeyi Technology Co. Ltd) were glow-discharged. A 4-μL aliquot of the sample was applied onto the grid for 1 min and stored at room temperature. Negative staining images were taken on an FEI Tecnai Spirit Bio TWIN microscope operating at 120 kV to examine the sample quality (Supplementary information, Figure S1B).

The same grids were used for cryo-EM sample preparation. Cryo-EM grids were prepared using Vitrobot Mark IV (FEI Company) at 8 °C and with 100% humidity. To increase the density of the spliceosomal particles and at the same time to reduce protein aggregation, a multiple-blotting method was adopted. Briefly, a 3-μL aliquot of the sample was loaded onto a glow-discharged copper grid coated with a thin carbon film. After 2 min, the protein solution was manually absorbed with the blotting paper and another 3-μl aliquot of the sample was loaded. These steps are repeated 3-4 times depending upon the sample concentration. Grids were then blotted by Vitrobot Mark IV (FEI Company) and rapidly plunged into liquid ethane cooled by liquid nitrogen.

Micrographs were collected using a Gatan K2 Summit detector (Gatan Company) mounted on a Titan Krios electron microscope (FEI Company) operating at 300-kV and equipped with a GIF Quantum energy filter (slit width 20 eV). Micrographs were recorded (Supplementary information, Figure S1C) in the super-resolution mode with a normal magnification of 105 000×, resulting in a calibrated pixel size of 0.669 Å. Each stack of 32 frames was exposed for 8 s, with an exposure time of 0.25 s per frame. The total dose rate was about 8.2 counts/second/physical-pixel (∼4.7 e^−^/s/Å^2^) for each stack. AutoEMation was used for the fully automated data collection^52^. All 32 frames in each stack were aligned and summed using the whole-image motion correction program MotionCor2^53^ and binned to a pixel size of 1.338 Å. The defocus value of each image was set from 0.8 to 1.8 μm and was determined by Gctf^54^.

### Image processing and calculation

Two data sets (I and II) prepared from different samples were used for the calculation (Supplementary information, Figures S2 and S3). Data set I is the same data as that described in the manuscript that reports the cryo-EM structure of the human spliceosomal C complex^24^. The synthetic pre-mRNA contains a 5′-exon, an intron, and a 3′-exon. Only a small proportion of the spliceosomal particles in this sample (named sample I, hereafter) are the B^act^ complex; the rest are the human C and C^*^ complexes. As will be made clear later, the B^act^ complexes in sample I have been identified to be the mature and late B^act^ complexes. Data set II is derived from the sample (named sample II hereafter) for which detailed purification procedure was described in Materials and Methods. The synthetic pre-mRNA in sample II is similar to that in sample I except that the 3′-exon has been deleted to prevent formation of any spliceosomes beyond the B^act^ complex. Consistent with the rationale, the spliceosomes derived from sample II are exclusively the early B^act^ complexes.

For data set I, 1 464 033 particles were auto-picked using the deep-learning program DeepPicker^55^. The convolutional neural network model for particle picking was trained using the previous data set of the ILS complex from *S. pombe*^9^. A guided multi-reference classification procedure was applied to the full data set using the program RELION2.0^56,57^ (Supplementary information, Figure S2). Details of this modified procedure were detailed in the manuscript reporting the cryo-EM structure of the human C^*^ complex^23^. Briefly, the generated 3D volumes of the human B^act^, C, and C^*^ complexes and four bad classes were obtained from a pilot analysis of 157 388 particles and were used as initial references (Round 1) (Supplementary information, Figure S2). These seven references were low-pass filtered to 40 Å. To avoid the problem of discarding good particles, we simultaneously performed three parallel multi-reference 3D classifications. Then, the particles that belong to the B^act^, C, and C^*^ complexes were combined and served as the input for a follow-up local classification. The particles that belong to the B^act^ complex (4.9%/5.1%/5.0% of the total particles in the three runs) were merged, and the duplicated particles were removed as described^5^. The remaining 113 931 particles, representing 7.8% of the original particles in data set I, gave an average resolution of 7.6 Å after auto-refinement with 2× binned particles (pixel size: 2.676 Å) (Supplementary information, Figure S2).

A second round (Round 2) of local 3D classification was performed for the remaining 113 931 particles. 2× binned particles (pixel size: 2.676 Å) were used for the classification (Supplementary information, Figure S2). A total of 49 218 particles from the good class (representing 43.2% of the input particles or 3.4% of the total original particles) yielded a reconstruction of the human B^act^ complex with an average resolution of 4.8 Å after auto-refinement using unbinned particles (pixel size: 1.338 Å).

In the final round (Round 3), the remaining 49 218 particles were classified without alignment but with a soft mask on the RNF113A region of the spliceosome. Two major classes, representing two different conformations, were identified. 27 405 particles (55.4% of the input particles) in one class yielded a reconstruction at an average resolution of 5.1 Å for the entire spliceosome, which was identified as the mature B^act^ complex. 14 316 particles (29.3% of the input particles) in the other class yielded a reconstruction at an average resolution of 6.5 Å for the entire spliceosome, which was identified as the late B^act^ complex (Supplementary information, Figures S2 and S4A; Tables S1 and S2).

For data set II, 629 472 particles were auto-picked using DeepPicker^55^. Similar to the processing of data set I, a guided multi-reference classification procedure was applied using RELION2.0^56^ (Supplementary information, Figure S3). The same set of seven references as used in the processing of data set I were used. To avoid the problem of discarding good particles, we simultaneously performed three parallel multi-reference 3D classifications. After the global classification, particles that belong to the B^act^ complex served as the input for a follow-up local classification. After local classification, the first three classes (references of B^act^, C, and C^*^) converged and became the B^act^ complexes. The particles that belong to the first three B^act^ complexes (7.4%/8.2%/5.6%, 6.3%/6.5%/7.0%, and 7.9%/8.1%/6.3% of the total particles in the three runs) were merged, and the duplicated particles were removed as described^5^. The remaining 186 780 particles represent 29.7% of the original particles in data set II, which gave rise to an average resolution of about 6.3 Å after auto-refinement with 2× binned particles (pixel size: 2.676 Å) (Supplementary information, Figure S3).

A second round (Round 2) of local 3D classification was performed on the remaining 186 780 particles. 2× binned particles (pixel size: 2.676 Å) were used for the classification (Supplementary information, Figure S3). A total of 96 523 particles from the good class (representing 51.7% of the input particles or 15.3% of the total original particles) yielded a reconstruction of the human B^act^ complex with an average resolution of 4.9 Å after auto-refinement using unbinned particles (pixel size: 1.338 Å) (Supplementary information, Figures S3 and S4A).

As will be clear from atomic modeling, the B^act^ complexes in sample I represent the mature and late states, whereas the B^act^ complexes in sample II exhibit an early state. Nonetheless, these three compositionally different B^act^ complexes share the same SF3b region. By combining these B^act^ particles from the two data sets (49 218 particles from data set I and 96 523 particles from data set II), we generated a larger date set of 145 741 particles. Following auto-refinement with a local mask on the SF3b region, the local resolution was improved to 4.2 Å (Supplementary information, Figures S3 and S4A; Table S2).

In the 4.9-Å cryo-EM map of the early B^act^ complex and the 4.8-Å cryo-EM map of the mature and late B^act^ complexes, the local resolution reaches 4.0-5.0 Å in the core of the spliceosome (Supplementary information, Figure S4B). The angular distributions of the particles used for the final reconstruction of both human B^act^ complexes are reasonable (Supplementary information, Figure S4C), and the refinement of the atomic coordinates did not suffer from severe over-fitting (Supplementary information, Figure S4D). The EM density maps of all three B^act^ complexes display similar overall structural features but with important differences in a number of key regions (Supplementary information, Figure S5). The density maps exhibit clear features for the secondary structural elements of the human B^act^ complex in the core region. The RNA elements and their interacting proteins are also reasonably well defined by the EM density maps and can be modeled with structural references from the human C^24^ and C^*^^23^ complexes and the yeast B^act^ complex^5^.

Reported resolutions were calculated on the basis of the FSC 0.143 criterion, and the FSC curves were corrected for the effects of a soft mask on the FSC curve using high-resolution noise substitution^58^. Prior to visualization, all density maps were corrected for the modulation transfer function of the detector, and then sharpened by applying a negative B-factor that was estimated using automated procedures^59^. Local resolution variations were estimated using ResMap^60^.

### Model building and refinement

Due to a wide range of resolution limits for the various regions of the human B^act^ complex, we combined homology modeling and rigid docking of components with known structures to generate an atomic model (Supplementary information, Table S2). Identification and docking of the components of the human B^act^ complex were facilitated by the atomic models of the human C^24^ and C^*^^23^ complex and the *S. cerevisiae* B^act^ complex^5^. The protein components that were derived from known structures of the protein data bank (PDB) are summarized in Supplementary information, Table S2. These structures were docked into the density map using COOT^61^ and fitted into density using CHIMERA^62^.

The atomic models of RNF113A and the N-terminal domain of SF3a66 in the human B^act^ complex were generated from Cwc24 and the N-terminal domain of Prp11 in the *S. cerevisiae* B^act^ complex^5^ using CHAINSAW^63^. The backbone was manually adjusted using COOT^61^. The atomic coordinates of U6 snRNA, protein components of the U5 and U2 snRNPs, protein components of the NTC and NTR complex, Prp17, and Aquarius from the human C^*^ complex (PDB code:5XJC^23^) were directly docked into the density maps of the human B^act^ complex and were manually adjusted using COOT^61^. Assignment of the 5-splice site (5′SS) and the duplex between U2 snRNA and the BPS was greatly aided by the structure of the yeast spliceosomal B^act^ complex^5^.

The crystal structure of the human SF3b core complex (PDB code: 5IFE^34^), including SF3b155, SF3b130, SF3b14b/PHF5A, and SF3b10, was docked into the density map guided by the yeast B^act^ structure^5^. SF3b145 was generated from Cus1 of the yeast B^act^ complex. The N-terminal domain of SF3b145 and the RRM domain of SF3b49 was generated from the crystal structure of Hsh49p in complex with Cus1p (PDB code: 5LSB^64^). Crystal structure of SF3b14a/p14 in complex with SF3b155 N-terminal fragments (PDB code: 2F9J^65^) was docked into the SF3b region of the cryo-EM maps. The crystal structure of the human SF3a complex (PDB code: 4DGW^66^), which includes SF3a120, SF3a66, and SF3a60, was docked into the extra density around the Sm ring of U2 snRNP. Prp2 is docked into the cryo-EM map on the basis of the structure of the yeast B^act^ complex^5^. CypE is docked into the map near the N-terminal HAT repeats of Syf1. Notably, a patch of weak EM density is also located in the same place of the yeast B^act^ complex as in the human B^act^ complex, suggesting that CypE may be recruited into the yeast B^act^ complex.

The final overall models of the early and mature B^act^ complexes were refined against the overall 4.9-Å and 4.8-Å map, respectively, using REFMAC in reciprocal space^67^, using secondary structure restraints that were generated by ProSMART^68^. The atomic model of the late B^act^ complex was generated by removing the RNF113A, NY-CO-10, and RNase H like domain of Prp8 from the mature B^act^ complex. Overfitting of the overall model was monitored by refining the model in one of the two independent maps from the gold-standard refinement approach, and testing the refined model against the other map^69^ (Supplementary information, Figure S4D). The structure of the human B^act^ complex was validated through examination of the Molprobity scores and statistics of the Ramachandran plots (Supplementary information, Table S1). Molprobity scores were calculated as described^70^. Distinguishing features of the cryo-EM maps among the three B^act^ complexes are detailed for the SF3b complex (Supplementary information, Figure S6), G10, Prp17, and RBM22 (Supplementary information, Figure S7), the N-terminal domain (NTD) of SF3a66 (Supplementary information, Figure S8), the NTC proteins (Supplementary information, Figure S9), RNF113A and NY-CO-10 (Supplementary information, Figure S10), and the SF3a and surrounding regions (Supplementary information, Figure S11).

### Accession code

The atomic coordinates for the early, mature and late B^act^ spliceosomes have been deposited in the Protein Data Bank with the accession code 5Z58, 5Z56 and 5Z57, respectively. The EM maps for the early, mature and late B^act^ spliceosomes have been deposited in EMDB with the accession code EMD-6891, EMD-6889 and EMD-6890, respectively.

## Author Contributions

XiaofengZ and XiechaoZ purified the human spliceosomal complexes and prepared the cryo-EM samples. XiaofengZ, XiechaoZ, JL, and CY collected the EM micrographs and processed the data. CY calculated the cryo-EM map and built the atomic model. All authors contributed to project discussion and structure analysis. XiaofengZ and XiechaoZ contributed to manuscript preparation. CY and YS wrote the manuscript. YS conceived and guided the project.

## Competing Financial Interests

The authors declare no competing financial interests.

## Supplementary Information

Supplementary information, Figure S1 Purification and EM analysis of the human spliceosomal B^act^ complex

Supplementary information, Figure S2 Analysis of the data set I for the cryo-EM reconstruction of the human mature and late B^act^ complexes

Supplementary information, Figure S3 Analysis of the data set II for the cryo-EM reconstruction of the human early B^act^ complex

Supplementary information, Figure S4 Cryo-EM analysis of the human spliceosomal B^act^ complex

Supplementary information, Figure S5 Comparison of the cryo-EM reconstructions for the early, the mature, and the late B^act^ complexes

Supplementary information, Figure S6 The cryo-EM density map of the SF3b complex in the B^act^ complex

Supplementary information, Figure S7 The cryo-EM density map of the NTR proteins and Prp17 in the early, mature, and late B^act^ complexes

Supplementary information, Figure S8 The cryo-EM density map of the SF3a complex components SF3a66 in the early, mature, and late B^act^ complexes

Supplementary information, Figure S9 The cryo-EM density maps of the NTC proteins in the early and mature B^act^ complexes

Supplementary information, Figure S10 The cryo-EM density map of the splicing factors NY-CO-10 and RNF113A in the early, mature, and late B^act^ complexes

Supplementary information, Figure S11 The cryo-EM density map of the SF3a complex in the mature B^act^ complex

Supplementary information, Figure S12 Structure of SF3b155 in the B^act^ complex

Supplementary information, Figure S13 Comparison of the RNA elements among the B complex, the B^act^ complex, and the C complex

Supplementary information, Table S1 Cryo-EM data collection and refinement statistics.

Supplementary information, Table S2 Summary of model building for the human spliceosomal B^act^ complex.

## References

[bib1] Wahl MC, Will CL, Luhrmann R. The spliceosome: design principles of a dynamic RNP machine. Cell 2009; 136:701–718. 19239890 10.1016/j.cell.2009.02.009

[bib2] Chan SP, Kao DI, Tsai WY, Cheng SC. The Prp19p-associated complex in spliceosome activation. Science 2003; 302:279–282. 12970570 10.1126/science.1086602

[bib3] Ohi MD, Gould KL. Characterization of interactions among the Cef1p-Prp19p-associated splicing complex. RNA 2002; 8:798–815. 12088152 10.1017/s1355838202025050 PMC1370298

[bib4] Fabrizio P, Dannenberg J, Dube P, et al. The evolutionarily conserved core design of the catalytic activation step of the yeast spliceosome. Mol Cell 2009; 36:593–608. 19941820 10.1016/j.molcel.2009.09.040

[bib5] Yan C, Wan R, Bai R, Huang G, Shi Y. Structure of a yeast activated spliceosome at 3.5 A resolution. Science 2016; 353:904–911. 27445306 10.1126/science.aag0291

[bib6] Rauhut R, Fabrizio P, Dybkov O, et al. Molecular architecture of the Saccharomyces cerevisiae activated spliceosome. Science 2016; 353:1399–1405. 27562955 10.1126/science.aag1906

[bib7] Shi Y. Mechanistic insights into precursor messenger RNA splicing by the spliceosome. Nat Rev Mol Cell Biol 2017; 18:655–670. 28951565 10.1038/nrm.2017.86

[bib8] Fica SM, Nagai K. Cryo-electron microscopy snapshots of the spliceosome: structural insights into a dynamic ribonucleoprotein machine. Nat Struct Mol Biol 2017; 24:791–799. 28981077 10.1038/nsmb.3463 PMC6386135

[bib9] Yan C, Hang J, Wan R, Huang M, Wong CC, Shi Y. Structure of a yeast spliceosome at 3.6-angstrom resolution. Science 2015; 349:1182–1191. 26292707 10.1126/science.aac7629

[bib10] Hang J, Wan R, Yan C, Shi Y. Structural basis of pre-mRNA splicing. Science 2015; 349:1191–1198. 26292705 10.1126/science.aac8159

[bib11] Shi Y. The spliceosome: a protein-directed metalloribozyme. J Mol Biol 2017; 429:2640–2653. 28733144 10.1016/j.jmb.2017.07.010

[bib12] Wan R, Yan C, Bai R, Huang G, Shi Y. Structure of a yeast catalytic step I spliceosome at 3.4 A resolution. Science 2016; 353:895–904. 27445308 10.1126/science.aag2235

[bib13] Galej WP, Wilkinson ME, Fica SM, Oubridge C, Newman AJ, Nagai K. Cryo-EM structure of the spliceosome immediately after branching. Nature 2016; 537:197–201. 27459055 10.1038/nature19316 PMC5156311

[bib14] Yan C, Wan R, Bai R, Huang G, Shi Y. Structure of a yeast step II catalytically activated spliceosome. Science 2017; 355:149–155. 27980089 10.1126/science.aak9979

[bib15] Fica SM, Oubridge C, Galej WP, et al. Structure of a spliceosome remodelled for exon ligation. Nature 2017; 542:377–380. 28076345 10.1038/nature21078 PMC5321579

[bib16] Wan R, Yan C, Bai R, Lei J, Shi Y. Structure of an intron lariat spliceosome from Saccharomyces cerevisiae. Cell 2017; 171:120–132.e12. 28919079 10.1016/j.cell.2017.08.029

[bib17] Bai R, Yan C, Wan R, Lei J, Shi Y. Structure of the Post-catalytic spliceosome from Saccharomyces cerevisiae. Cell 2017; 171:1589–1598.e8. 29153833 10.1016/j.cell.2017.10.038

[bib18] Liu S, Li X, Zhang L, et al. Structure of the yeast spliceosomal postcatalytic P complex. Science 2017; 358:1278–1283. 29146870 10.1126/science.aar3462 PMC5828012

[bib19] Wilkinson ME, Fica SM, Galej WP, Norman CM, Newman AJ, Nagai K. Postcatalytic spliceosome structure reveals mechanism of 3′-splice site selection. Science 2017; 358:1283–1288. 29146871 10.1126/science.aar3729 PMC5808836

[bib20] Plaschka C, Lin PC, Nagai K. Structure of a pre-catalytic spliceosome. Nature 2017; 546:617–621. 28530653 10.1038/nature22799 PMC5503131

[bib21] Bertram K, Agafonov DE, Dybkov O, et al. Cryo-EM structure of a pre-catalytic human spliceosome primed for activation. Cell 2017; 170:701–713.e711. 28781166 10.1016/j.cell.2017.07.011

[bib22] Bertram K, Agafonov DE, Liu WT, et al. Cryo-EM structure of a human spliceosome activated for step 2 of splicing. Nature 2017; 542:318–323. 28076346 10.1038/nature21079

[bib23] Zhang X, Yan C, Hang J, Finci LI, Lei J, Shi Y. An atomic structure of the human spliceosome. Cell 2017; 169:918–929.e914. 28502770 10.1016/j.cell.2017.04.033

[bib24] Zhan X, Yan C, Zhang X, Lei J, Shi Y. Structure of a human catalytic step I spliceosome. Science 2018; doi: 10.1126/science.aar6401. 10.1126/science.aar6401 29301961

[bib25] Cheng SC. Formation of the yeast splicing complex A1 and association of the splicing factor PRP19 with the pre-mRNA are independent of the 3′ region of the intron. Nucleic Acids Res 1994; 22:1548–1554. 8202353 10.1093/nar/22.9.1548 PMC308028

[bib26] Rasche N, Dybkov O, Schmitzova J, Akyildiz B, Fabrizio P, Luhrmann R. Cwc2 and its human homologue RBM22 promote an active conformation of the spliceosome catalytic centre. EMBO J 2012; 31:1591–1604. 22246180 10.1038/emboj.2011.502 PMC3321175

[bib27] Ohrt T, Prior M, Dannenberg J, et al. Prp2-mediated protein rearrangements at the catalytic core of the spliceosome as revealed by dcFCCS. RNA 2012; 18:1244–1256. 22535589 10.1261/rna.033316.112 PMC3358646

[bib28] Wu NY, Chung CS, Cheng SC. Role of Cwc24 in the first catalytic step of splicing and fidelity of 5′ splice site selection. Mol Cell Biol 2017; 37:pii: e00580–16. 27994011 10.1128/MCB.00580-16 PMC5335513

[bib29] Golas MM, Sander B, Will CL, Luhrmann R, Stark H. Molecular architecture of the multiprotein splicing factor SF3b. Science 2003; 300:980–984. 12738865 10.1126/science.1084155

[bib30] Gozani O, Feld R, Reed R. Evidence that sequence-independent binding of highly conserved U2 snRNP proteins upstream of the branch site is required for assembly of spliceosomal complex A. Genes Dev 1996; 10:233–243. 8566756 10.1101/gad.10.2.233

[bib31] Query CC, McCaw PS, Sharp PA. A minimal spliceosomal complex A recognizes the branch site and polypyrimidine tract. Mol Cell Biol 1997; 17:2944–2953. 9111366 10.1128/mcb.17.5.2944 PMC232146

[bib32] Will CL, Urlaub H, Achsel T, Gentzel M, Wilm M, Luhrmann R. Characterization of novel SF3b and 17S U2 snRNP proteins, including a human Prp5p homologue and an SF3b DEAD-box protein. EMBO J 2002; 21:4978–4988. 12234937 10.1093/emboj/cdf480 PMC126279

[bib33] MacMillan AM, Query CC, Allerson CR, Chen S, Verdine GL, Sharp PA. Dynamic association of proteins with the pre-mRNA branch region. Genes Dev 1994; 8:3008–3020. 8001820 10.1101/gad.8.24.3008

[bib34] Cretu C, Schmitzova J, Ponce-Salvatierra A, et al. Molecular architecture of SF3b and structural consequences of its cancer-related mutations. Mol Cell 2016; 64:307–319. 27720643 10.1016/j.molcel.2016.08.036

[bib35] Query CC, Strobel SA, Sharp PA. Three recognition events at the branch-site adenine. EMBO J 1996; 15:1392–1402. 8635472 PMC450044

[bib36] Dziembowski A, Ventura AP, Rutz B, et al. Proteomic analysis identifies a new complex required for nuclear pre-mRNA retention and splicing. EMBO J 2004; 23:4847–4856. 15565172 10.1038/sj.emboj.7600482 PMC535094

[bib37] Wysoczanski P, Schneider C, Xiang S, et al. Cooperative structure of the heterotrimeric pre-mRNA retention and splicing complex. Nat Struct Mol Biol 2014; 21:911–918. 25218446 10.1038/nsmb.2889

[bib38] Ohi MD, Link AJ, Ren L, Jennings JL, McDonald WH, Gould KL. Proteomics analysis reveals stable multiprotein complexes in both fission and budding yeasts containing Myb-related Cdc5p/Cef1p, novel pre-mRNA splicing factors, and snRNAs. Mol Cell Biol 2002; 22:2011–2024. 11884590 10.1128/MCB.22.7.2011-2024.2002 PMC133674

[bib39] Raghunathan PL, Guthrie C. RNA unwinding in U4/U6 snRNPs requires ATP hydrolysis and the DEIH-box splicing factor Brr2. Curr Biol 1998; 8:847–855. 9705931 10.1016/s0960-9822(07)00345-4

[bib40] Laggerbauer B, Achsel T, Luhrmann R. The human U5–200kD DEXH-box protein unwinds U4/U6 RNA duplices *in vitro*. Proc Natl Acad Sci USA 1998; 95:4188–4192. 9539711 10.1073/pnas.95.8.4188 PMC22463

[bib41] Kim SH, Lin RJ. Spliceosome activation by PRP2 ATPase prior to the first transesterification reaction of pre-mRNA splicing. Mol Cell Biol 1996; 16:6810–6819. 8943336 10.1128/mcb.16.12.6810 PMC231684

[bib42] Schwer B, Guthrie C. A conformational rearrangement in the spliceosome is dependent on PRP16 and ATP hydrolysis. EMBO J 1992; 11:5033–5039. 1464325 10.1002/j.1460-2075.1992.tb05610.x PMC556981

[bib43] Schwer B. A conformational rearrangement in the spliceosome sets the stage for Prp22-dependent mRNA release. Mol Cell 2008; 30:743–754. 18570877 10.1016/j.molcel.2008.05.003 PMC2465764

[bib44] Company M, Arenas J, Abelson J. Requirement of the RNA helicase-like protein PRP22 for release of messenger RNA from spliceosomes. Nature 1991; 349:487–493. 1992352 10.1038/349487a0

[bib45] Zavanelli MI, Ares M, Jr. Efficient association of U2 snRNPs with pre-mRNA requires an essential U2 RNA structural element. Genes Dev 1991; 5:2521–2533. 1752442 10.1101/gad.5.12b.2521

[bib46] Hilliker AK, Mefford MA, Staley JP. U2 toggles iteratively between the stem IIa and stem IIc conformations to promote pre-mRNA splicing. Genes Dev 2007; 21:821–834. 17403782 10.1101/gad.1536107 PMC1838533

[bib47] Perriman RJ, Ares M, Jr. Rearrangement of competing U2 RNA helices within the spliceosome promotes multiple steps in splicing. Genes Dev 2007; 21:811–820. 17403781 10.1101/gad.1524307 PMC1838532

[bib48] Bon E, Casaregola S, Blandin G, et al. Molecular evolution of eukaryotic genomes: hemiascomycetous yeast spliceosomal introns. Nucleic Acids Res 2003; 31:1121–1135. 12582231 10.1093/nar/gkg213 PMC150231

[bib49] Bessonov S, Anokhina M, Krasauskas A, et al. Characterization of purified human B^act^ spliceosomal complexes reveals compositional and morphological changes during spliceosome activation and first step catalysis. RNA 2010; 16:2384–2403. 20980672 10.1261/rna.2456210 PMC2995400

[bib50] Dignam JD, Lebovitz RM, Roeder RG. Accurate transcription initiation by RNA polymerase II in a soluble extract from isolated mammalian nuclei. Nucleic Acids Res 1983; 11:1475–1489. 6828386 10.1093/nar/11.5.1475 PMC325809

[bib51] Kastner B, Fischer N, Golas MM, et al. GraFix: sample preparation for single-particle electron cryomicroscopy. Nat Methods 2008; 5:53–55. 18157137 10.1038/nmeth1139

[bib52] Lei J, Frank J. Automated acquisition of cryo-electron micrographs for single particle reconstruction on an FEI Tecnai electron microscope. J Struct Biol 2005; 150:69–80. 15797731 10.1016/j.jsb.2005.01.002

[bib53] Zheng SQ, Palovcak E, Armache JP, Verba KA, Cheng Y, Agard DA. MotionCor2: anisotropic correction of beam-induced motion for improved cryo-electron microscopy. Nat Methods 2017; 14:331–332. 28250466 10.1038/nmeth.4193 PMC5494038

[bib54] Zhang K. Gctf: Real-time CTF determination and correction. J Struct Biol 2016; 193:1–12. 26592709 10.1016/j.jsb.2015.11.003 PMC4711343

[bib55] Wang F, Gong H, Liu G, et al. DeepPicker: A deep learning approach for fully automated particle picking in cryo-EM. J Struct Biol 2016; 195:325–336. 27424268 10.1016/j.jsb.2016.07.006

[bib56] Kimanius D, Forsberg BO, Scheres SH, Lindahl E. Accelerated cryo-EM structure determination with parallelisation using GPUs in RELION-2. eLife 2016; 5:pii: e18722. 27845625 10.7554/eLife.18722 PMC5310839

[bib57] Scheres SH. RELION: implementation of a Bayesian approach to cryo-EM structure determination. J Struct Biol 2012; 180:519–530. 23000701 10.1016/j.jsb.2012.09.006 PMC3690530

[bib58] Chen S, McMullan G, Faruqi AR, et al. High-resolution noise substitution to measure overfitting and validate resolution in 3D structure determination by single particle electron cryomicroscopy. Ultramicroscopy 2013; 135:24–35. 23872039 10.1016/j.ultramic.2013.06.004 PMC3834153

[bib59] Rosenthal PB, Henderson R. Optimal determination of particle orientation, absolute hand, and contrast loss in single-particle electron cryomicroscopy. J Mol Biol 2003; 333:721–745. 14568533 10.1016/j.jmb.2003.07.013

[bib60] Kucukelbir A, Sigworth FJ, Tagare HD. Quantifying the local resolution of cryo-EM density maps. Nat Methods 2014; 11:63–65. 24213166 10.1038/nmeth.2727 PMC3903095

[bib61] Emsley P, Cowtan K. Coot: model-building tools for molecular graphics. Acta Crystallogr D Biol Crystallogr 2004; 60:2126–2132. 15572765 10.1107/S0907444904019158

[bib62] Pettersen EF, Goddard TD, Huang CC, et al. UCSF Chimera--a visualization system for exploratory research and analysis. J Comput Chem 2004; 25:1605–1612. 15264254 10.1002/jcc.20084

[bib63] Stein N. CHAINSAW: a program for mutating pdb files used as templates in molecular replacement. J Appl Crystallogr 2008; 41:641–643.

[bib64] van Roon AM, Oubridge C, Obayashi E, et al. Crystal structure of U2 snRNP SF3b components: Hsh49p in complex with Cus1p-binding domain. RNA 2017; 23:968–981. 28348170 10.1261/rna.059378.116 PMC5435868

[bib65] Schellenberg MJ, Edwards RA, Ritchie DB, et al. Crystal structure of a core spliceosomal protein interface. Proc Natl Acad Sci USA 2006; 103:1266–1271. 16432215 10.1073/pnas.0508048103 PMC1360545

[bib66] Lin PC, Xu RM. Structure and assembly of the SF3a splicing factor complex of U2 snRNP. EMBO J 2012; 31:1579–1590. 22314233 10.1038/emboj.2012.7 PMC3321192

[bib67] Murshudov GN, Vagin AA, Dodson EJ. Refinement of macromolecular structures by the maximum-likelihood method. Acta Crystallogr D Biol Crystallogr 1997; 53:240–255. 15299926 10.1107/S0907444996012255

[bib68] Nicholls RA, Fischer M, McNicholas S, Murshudov GN. Conformation-independent structural comparison of macromolecules with ProSMART. Acta Crystallogr D Biol Crystallogr 2014; 70:2487–2499. 25195761 10.1107/S1399004714016241 PMC4157452

[bib69] Amunts A, Brown A, Bai XC, et al. Structure of the yeast mitochondrial large ribosomal subunit. Science 2014; 343:1485–1489. 24675956 10.1126/science.1249410 PMC4046073

[bib70] Davis IW, Leaver-Fay A, Chen VB, et al. MolProbity: all-atom contacts and structure validation for proteins and nucleic acids. Nucleic Acids Res 2007; 35:W375–383. 17452350 10.1093/nar/gkm216 PMC1933162

