# Supplementary material for: Structure of the human activated spliceosome in three conformational states
Source: Cell Res. 2018 Jan 23;28(3):307–22. doi: 10.1038/cr.2018.14 (PMC5835773; doi:10.1038/cr.2018.14)
Supplement: Supplementary information, Figure S1 — Purification and EM analysis of the human spliceosomal Bact complex [file cr201814x1.pdf]

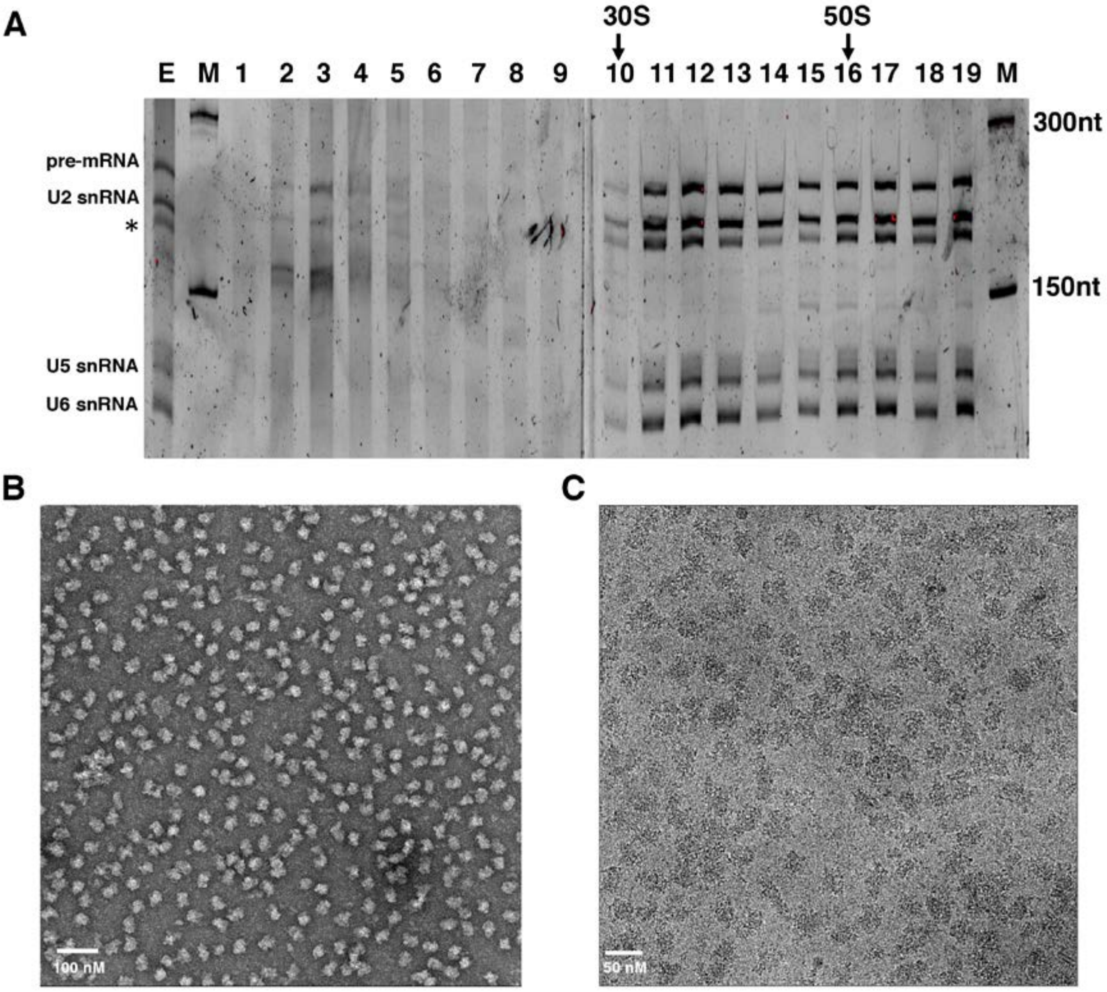

Figure S1 Purification and EM analysis of the human spliceosomal B^{act} complex. (**A**) The affinity-purified human spliceosomes were fractionated by glycerol gradient centrifugation. The total RNA from each fraction was electrophoresed in a denaturing Urea-PAGE gel, followed by SYBR® Gold staining. Fractions 11-15 may contain the human spliceosomal B^{act} complex and were pooled for the preparation of cryo-EM sample. (**B**) A negative-staining (by uranyl acetate) electron microscopy (EM) micrograph of the final sample. Scale bar, 100 nm. (**C**) A representative cryo-EM micrograph of the final sample. Scale bar, 50 nm.
