# Supplementary material for: Structure of the human activated spliceosome in three conformational states
Source: Cell Res. 2018 Jan 23;28(3):307–22. doi: 10.1038/cr.2018.14 (PMC5835773; doi:10.1038/cr.2018.14)
Supplement: Supplementary information, Figure S2 — Analysis of the data set I for the cryo-EM reconstruction of the human mature and late Bact complexes [file cr201814x2.pdf]

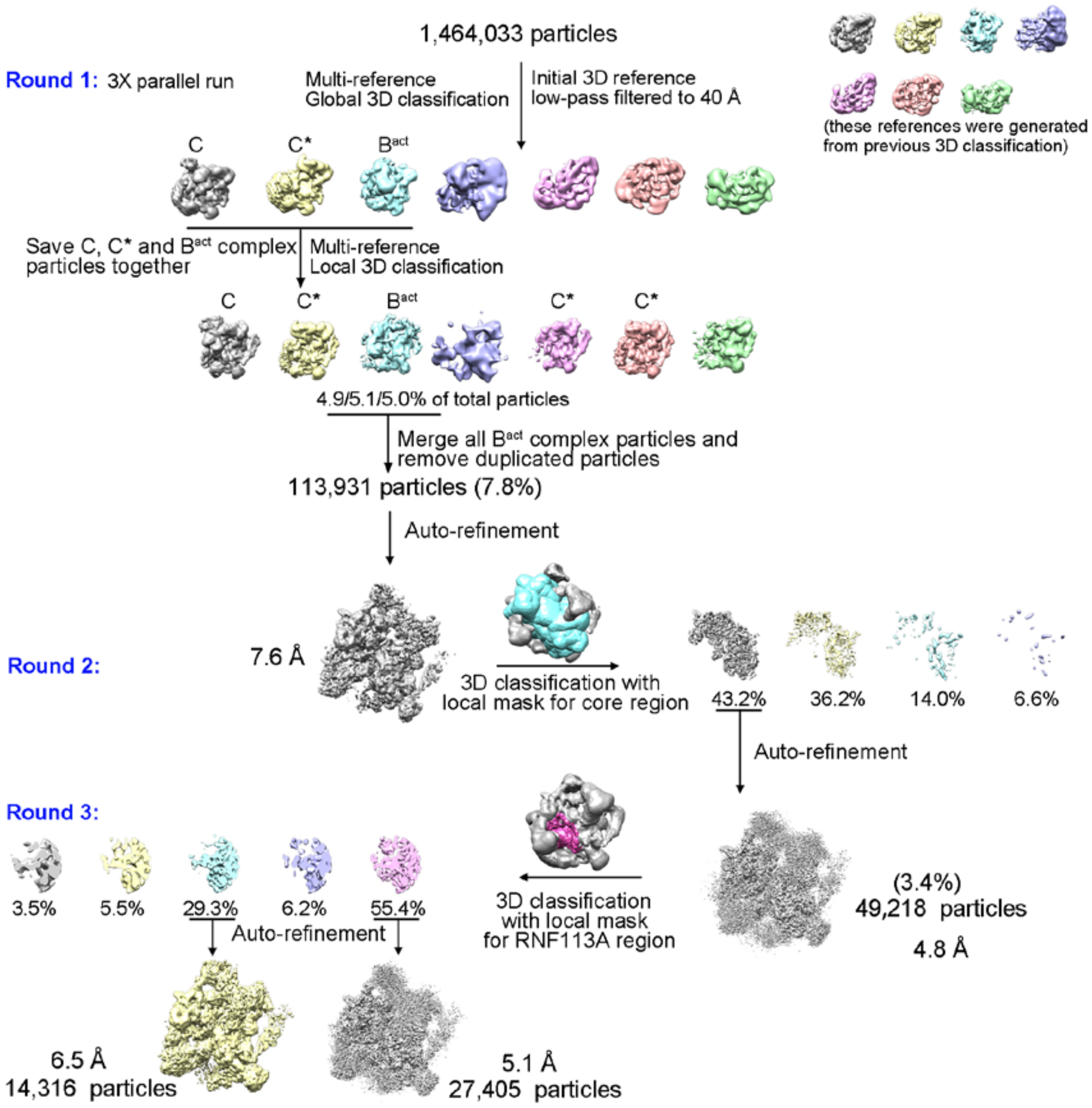

Figure S2 Analysis of the data set I for the cryo-EM reconstruction of the human mature and late B^{act} complexes. Shown here is a flow chart for the analysis of the data set I of 1,464,033 particles of the human spliceosomal complexes. There are three distinct spliceosomal complexes – the B^{act}, C, and C^{*}– in the sample (sample I). The analysis focused on the particles that represent the B^{act} complex. Please refer to Materials and Methods for details.
