# Supplementary material for: Structure of the human activated spliceosome in three conformational states
Source: Cell Res. 2018 Jan 23;28(3):307–22. doi: 10.1038/cr.2018.14 (PMC5835773; doi:10.1038/cr.2018.14)
Supplement: Supplementary information, Figure S3 — Analysis of the data set II for the cryo-EM reconstruction of the human early Bact complex [file cr201814x3.pdf]

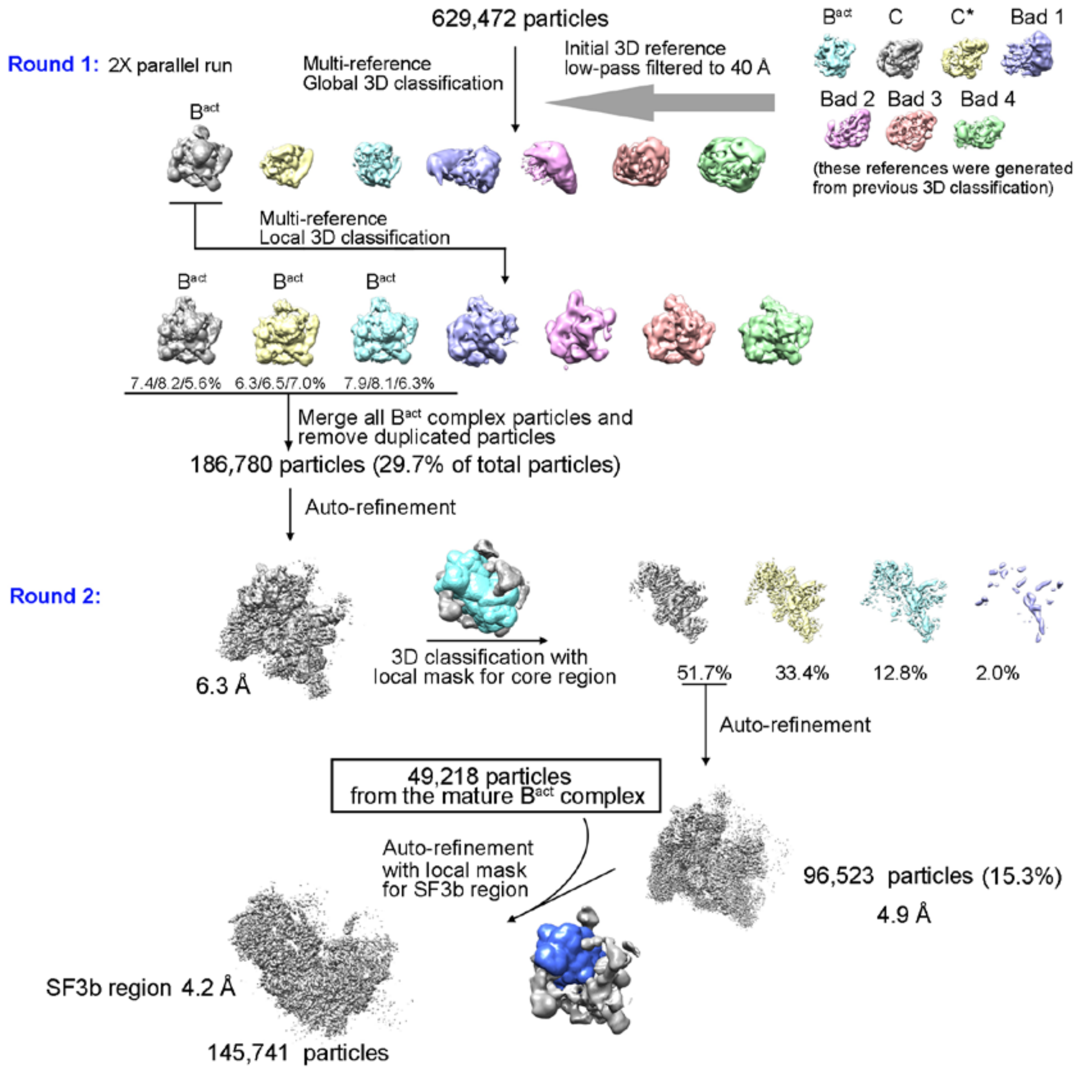

Figure S3 Analysis of the data set II for the cryo-EM reconstruction of the human early B^{act} complex. Shown here is a flow chart for the cryo-EM data processing and structure determination of the human spliceosomal early B^{act} complex. Compared to the mature and late B^{act} complexes derived from data set I, the B^{act} complex captured here may represent that of an early stage. The final reconstruction has an average resolution of 4.9 Å. Please refer to Materials and Methods for details.
