# Supplementary material for: Structure of the human activated spliceosome in three conformational states
Source: Cell Res. 2018 Jan 23;28(3):307–22. doi: 10.1038/cr.2018.14 (PMC5835773; doi:10.1038/cr.2018.14)
Supplement: Supplementary information, Figure S4 — Cryo-EM analysis of the human spliceosomal Bact complex [file cr201814x4.pdf]

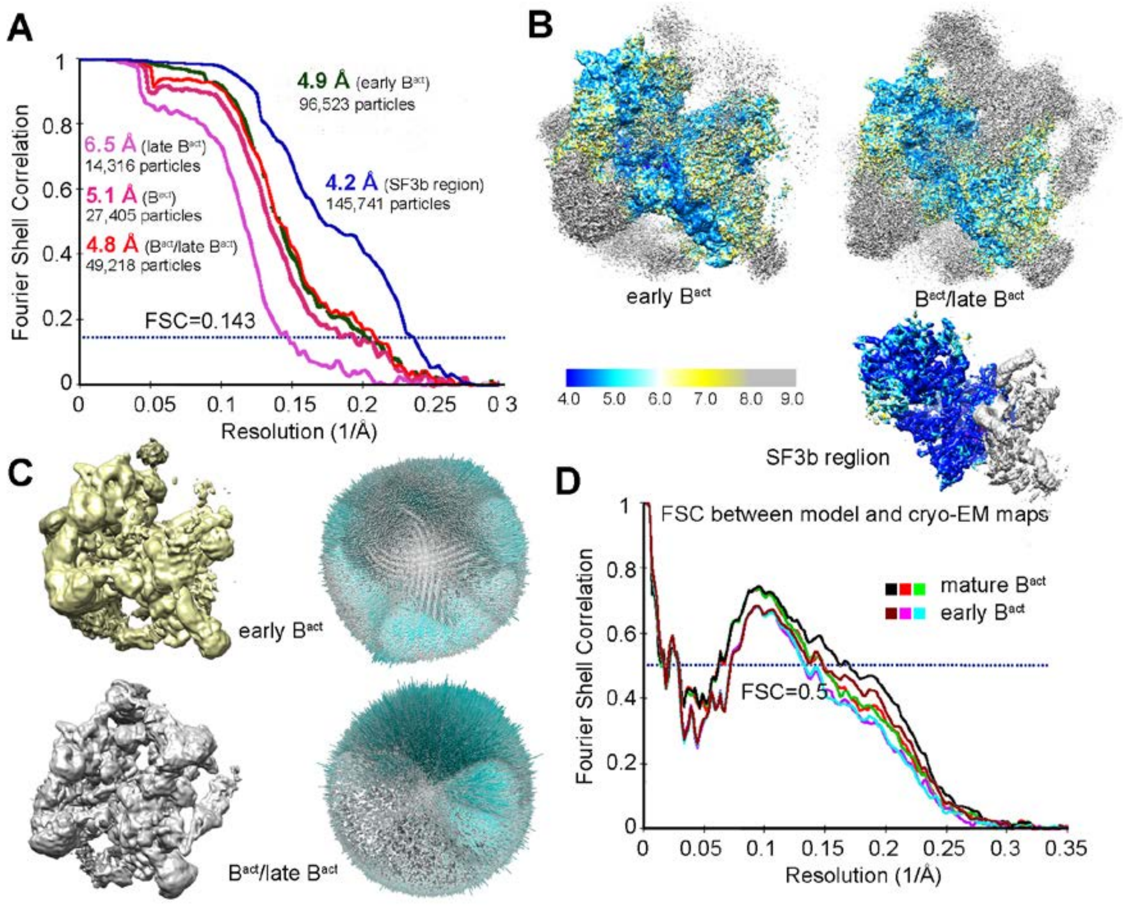

Figure S4 Cryo-EM analysis of the human spliceosomal B^{act} complex. (**A**) The average resolutions of the overall map for early, mature, and late B^{act} complexes are estimated to be 4.9 Å, 5.1 Å and 6.5 Å, respectively, on the basis of the FSC value of 0.143. The average resolution of the overall map for the mature and late B^{act} complexes is estimated to be 4.8 Å on the basis of 49,218 particles. The average resolutions of the local map for the SF3b region is estimated to be 4.2 Å on the basis of 145,741 particles. (**B**) The local resolutions are color-coded for the early B^{act} complex (left panel), the mature and late B^{act} complexes (upper right panel), and he SF3b region (lower right panel). (**C**) Angular distributions of the particles used for the reconstruction of the early B^{act} complex (upper right panel) and the mature and late B^{act} complexes (lower right panel). Each cylinder represents one view and

the height of the cylinder is proportional to the number of particles for that view. The corresponding EM density maps for the early and mature/late B^{act} complexes are shown in the upper left and lower left panels, respectively. (**D**) The FSC curves of the final refined model versus the overall 4.8/4.9 Å map it was refined against (black/chocolate); of the model refined in the first of the two independent maps used for the gold-standard FSC versus that same map (red/magenta); and of the model refined in the first of the two independent maps versus the second independent map (green/cyan). The generally similar appearances between the red and green curves indicates that the refinement of the atomic coordinates did not suffer from severe over-fitting.
