# Supplementary material for: Structure of the human activated spliceosome in three conformational states
Source: Cell Res. 2018 Jan 23;28(3):307–22. doi: 10.1038/cr.2018.14 (PMC5835773; doi:10.1038/cr.2018.14)
Supplement: Supplementary information, Figure S5 — Comparison of the cryo-EM reconstructions for the early, the mature, and the late Bact complexes [file cr201814x5.pdf]

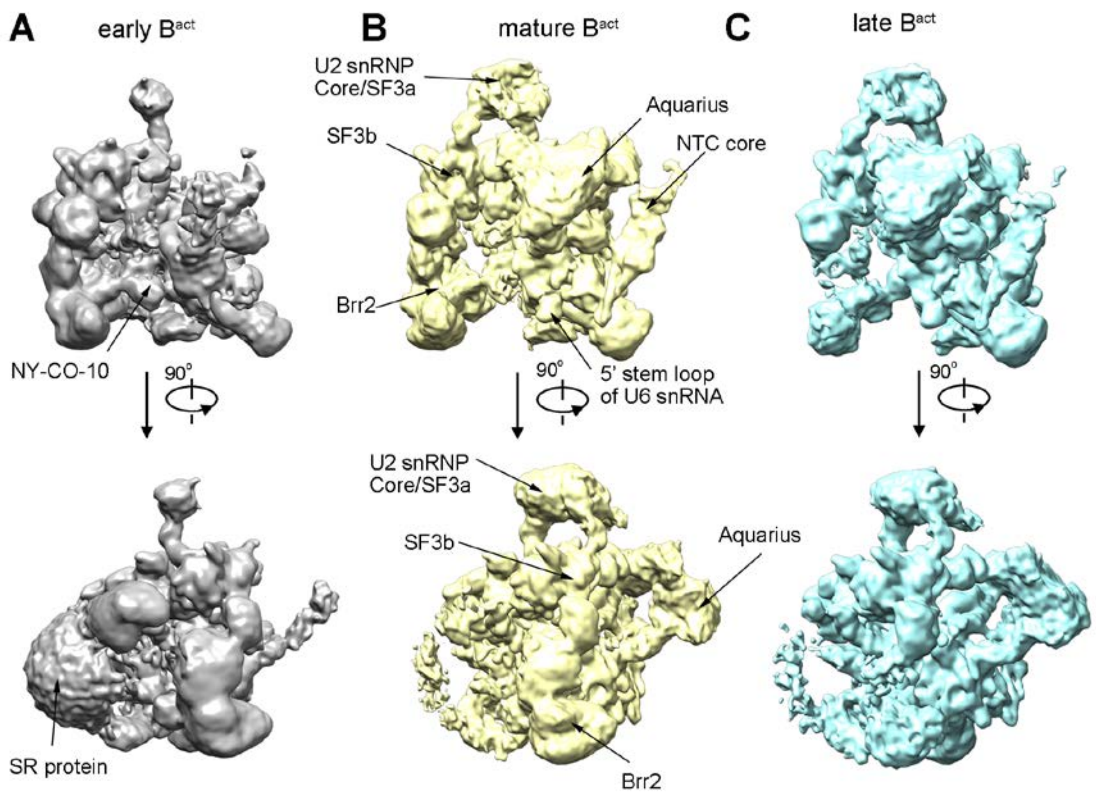

Figure S5 Comparison of the cryo-EM reconstructions for the early, the mature, and the late B^{act} complexes. (**A**) The cryo-EM map of the early B^{act} complex (gray). Two perpendicular view around the vertical axis are shown. (**B**) The cryo-EM map of the mature B^{act} complex (pale yellow). (**C**) The cryo-EM map of the late B^{act} complex (pale cyan). All three maps are low-pass filtered to approximately 10 Å. The three cryo-EM maps are displayed in the same orientation with their U5 snRNP regions aligned. To facilitate comparison, some of the characteristic regions are indicated by black arrows.
