# Supplementary material for: Structure of the human activated spliceosome in three conformational states
Source: Cell Res. 2018 Jan 23;28(3):307–22. doi: 10.1038/cr.2018.14 (PMC5835773; doi:10.1038/cr.2018.14)
Supplement: Supplementary information, Figure S6 — The cryo-EM density map of the SF3b complex in the Bact complex [file cr201814x6.pdf]

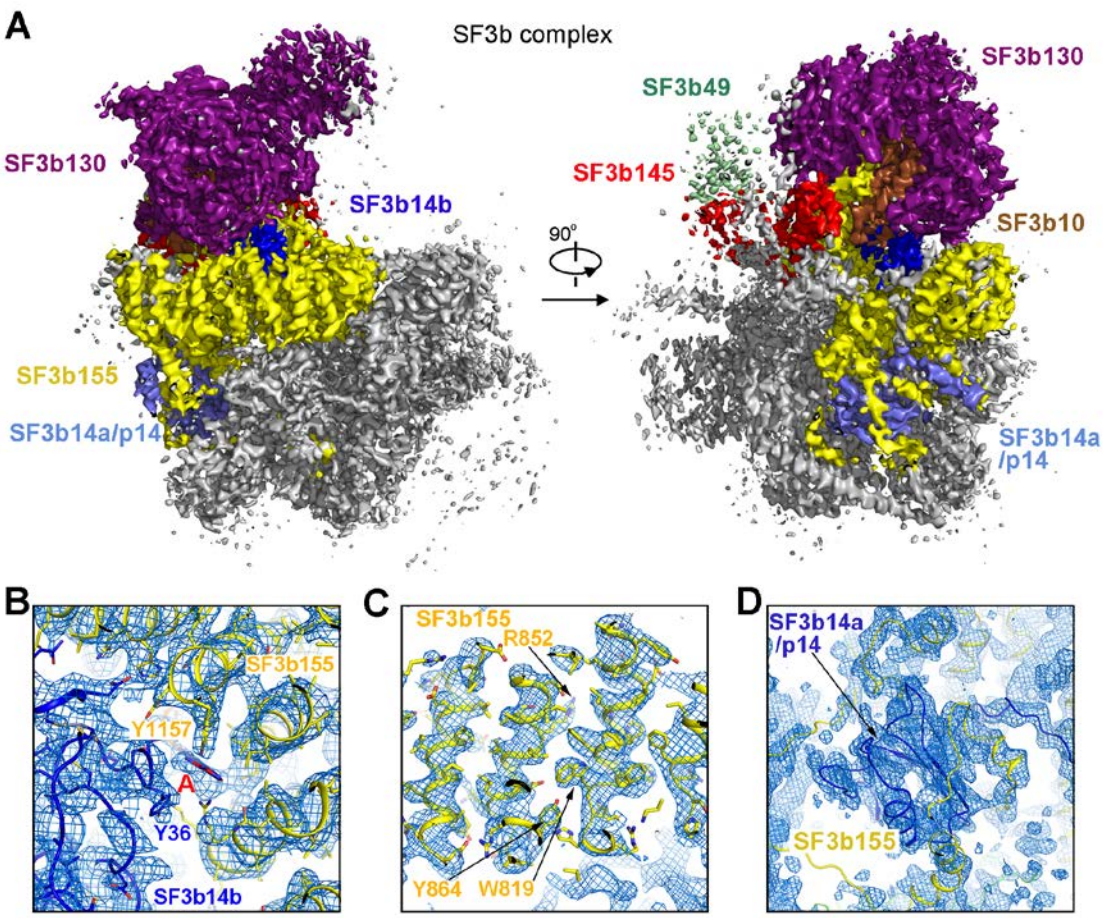


**Figure S6** The cryo-EM density map of the SF3b complex in the $B^{act}$ complex. (**A**) The cryo-EM density map of the SF3b region at 4.2 Å resolution. The components of the SF3b complex are color-coded: SF3b130 (dark purple), SF3b155 (yellow), SF3b145 (red), SF3b14a/p13 (slate), SF3b14b/PHF5A (blue), SF3b10b (chocolate) and SF3b49 (smudge). The density for SF3b49 is poor at the contour level displayed here. (**B**) A close-up view of the 4.2-Å resolution EM density map on the region centered around the nucleophile-containing adenine base of the BPS. The adenine base is surrounded by a hydrophobic pocket formed by residues from SF3b155 and SF3b14b. (**C**) A close-up view of the 4.2-Å resolution EM density map on representative HEAT repeats of SF3b155. The side chains of bulky residues can be visualized. (**D**) A close-up view of the 4.2-Å EM density map on SF3b14a/p14. SF3b14a/p14 is surrounded by a loop from SF3b155.
