# Supplementary material for: Structure of the human activated spliceosome in three conformational states
Source: Cell Res. 2018 Jan 23;28(3):307–22. doi: 10.1038/cr.2018.14 (PMC5835773; doi:10.1038/cr.2018.14)
Supplement: Supplementary information, Figure S7 — The cryo-EM density map of the NTR proteins and Prp17 in the early, mature, and late Bact complexes [file cr201814x7.pdf]

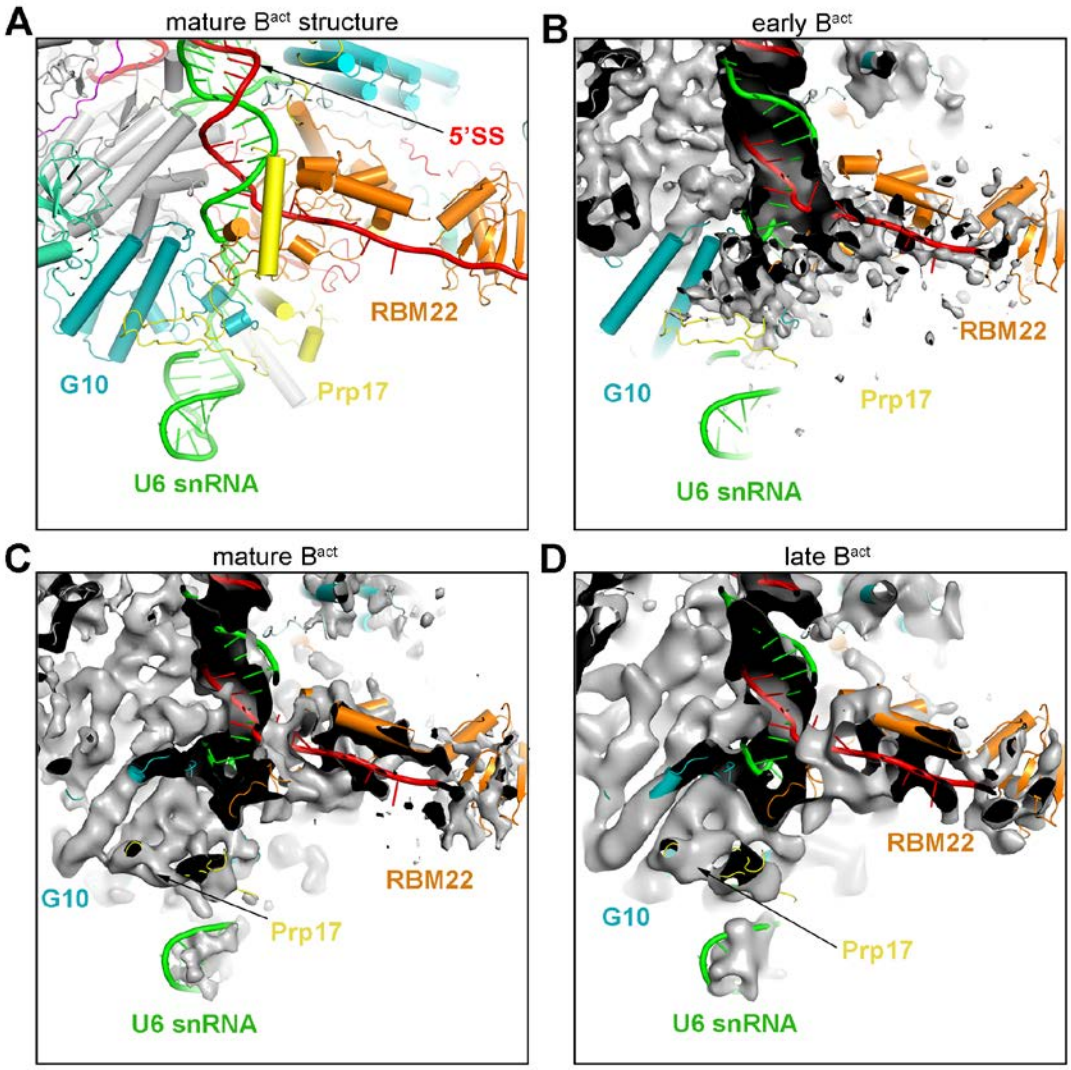

Figure S7 The cryo-EM density map of the NTR proteins and Prp17 in the early, mature, and late B^{act} complexes. (**A**) The structure of the mature B^{act} complex around the U6 snRNA/5'SS duplex region. Prp17 (yellow) and the NTR components G10 (teal) and RBM22 (orange) are highlighted here. (**B**) A section of the 4.9-Å resolution EM density map of the early B^{act} complex around the U6 snRNA/5'SS region. There is no obvious density for Prp17, G10, or RBM22. Consequently, the 5'-stem loop of the U6 snRNA is also flexible in the absence of these three binding proteins. (**C**) A section of the 5.1-Å resolution EM density map of the mature B^{act} complex around the U6 snRNA/5'SS region. In contrast to that of the early B^{act} complex, the density is strong for Prp17 and the NTR components G10 and RBM22.

(**D**) A section of the 6.5-Å resolution EM density map of the late B^{act} complex around the U6 snRNA/5’SS region. The density in this region is similarly strong as that in the mature B^{act} complex.
