# Supplementary material for: Structure of the human activated spliceosome in three conformational states
Source: Cell Res. 2018 Jan 23;28(3):307–22. doi: 10.1038/cr.2018.14 (PMC5835773; doi:10.1038/cr.2018.14)
Supplement: Supplementary information, Figure S8 — The cryo-EM density map of the SF3a complex components SF3a66 in the early, mature, and late Bact complexes [file cr201814x8.pdf]

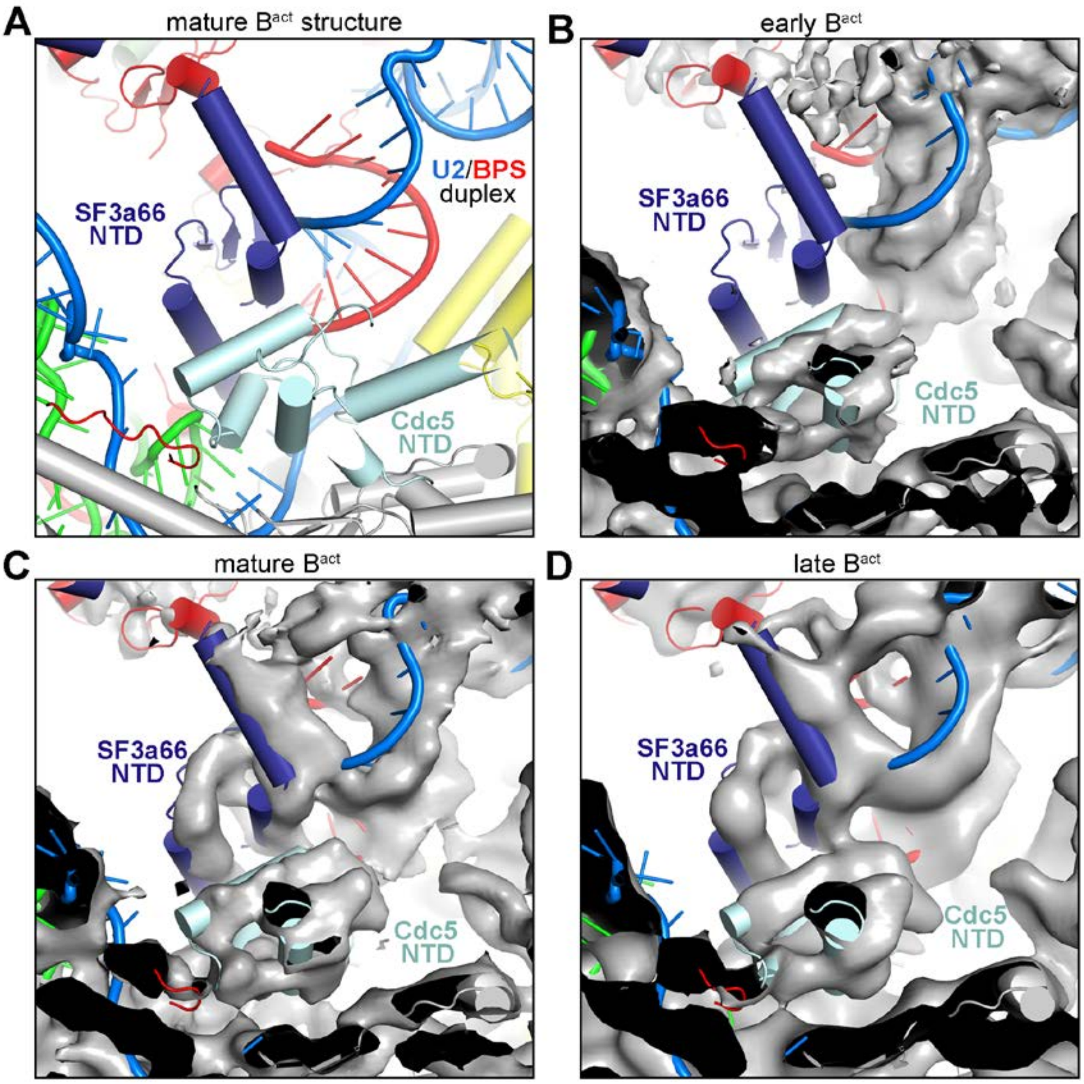

Figure S8 The cryo-EM density map of the SF3a complex components SF3a66 in the early, mature, and late B^{act} complexes. (**A**) The structure of the N-terminal domain (NTD) of SF3a66 in the mature B^{act} complex. The NTD of SF3a66 is located close to the U2 snRNA/BPS duplex at the 5'-end of the BPS. (**B**) A section of the 4.9-Å resolution EM density map of the SF3a66 NTD in the early B^{act} complex. There is no obvious density for the NTD of SF3a66. (**C**) A section of the 5.1-Å resolution EM density map of the SF3a66 NTD in the mature B^{act} complex. Strong density is present for the NTD of SF3a66. The association of the NTD of SF3a66 with the U2 snRNA/BPS duplex may stabilize the interaction between the SF3a complex and the U2 snRNP core, thus indirectly bridging the interaction of the

U2 snRNP with the NTC proteins. (**D**) A section of the 6.5-Å resolution EM density map of the SF3a66 NTD in the late B^{act} complex. The density for the NTD of SF3a66 is similarly strong as that in the mature B^{act} complex.
