# Supplementary material for: Structure of the human activated spliceosome in three conformational states
Source: Cell Res. 2018 Jan 23;28(3):307–22. doi: 10.1038/cr.2018.14 (PMC5835773; doi:10.1038/cr.2018.14)
Supplement: Supplementary information, Figure S9 — The cryo-EM density maps of the NTC proteins in the early and mature Bact complexes [file cr201814x9.pdf]

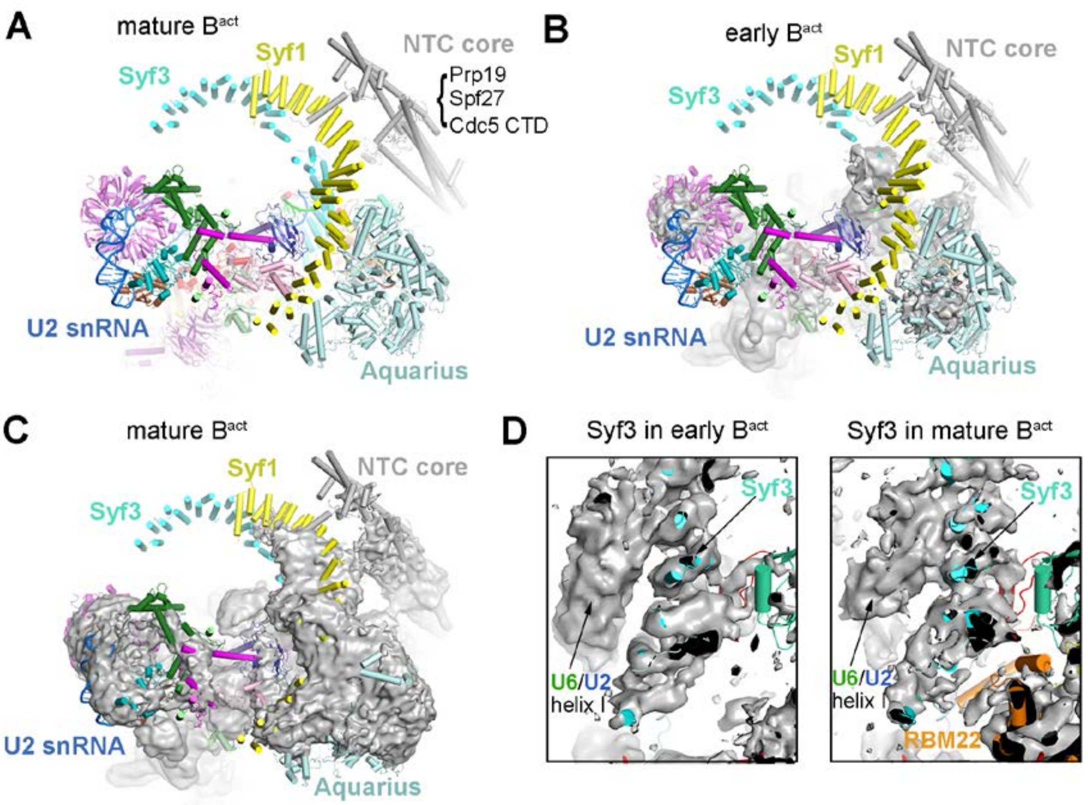

Figure S9 The cryo-EM density maps of the NTC proteins in the early and mature B^{act} complexes. (**A**) The local structure of the mature B^{act} complex around the regions of the U2 snRNP, the NTC proteins, and Aquarius. All components are color-coded except the NTC core, which includes Prp19, Spf27, and the C-terminal domain (CTD) of Cdc5. (**B**) The local structure from panel A is shown in the context of the 4.9-Å resolution cryo-EM density map of the early B^{act} complex. The early and mature B^{act} complexes are perfectly superimposed; but for clarity, only the structure of the mature B^{act} complex is shown here. Most of the NTC proteins (including Syf3 and the NTC core), display little or no density in the map and are absent from the early B^{act} complex. (**C**) The local structure from panel A is shown in the context of the 5.1-Å resolution EM density map of the mature B^{act} complex. The contour level of the EM map is the same as that in panel B. The NTC proteins in the mature B^{act} complex display strong density in the map and can be assigned. (**D**) A close-up view on the cryo-EM maps of the early and mature B^{act} complexes around

the N-terminal region of the NTC component Syf3. In both maps, the N-terminal region of Syf3 is clearly identified by strong density. This analysis indicates that Syf3 is already recruited into the early B^{act} complex but its C-terminal region is likely flexible in the absence of surrounding interacting proteins (as suggested by the weak density for this region).
