# Supplementary material for: Structure of the human activated spliceosome in three conformational states
Source: Cell Res. 2018 Jan 23;28(3):307–22. doi: 10.1038/cr.2018.14 (PMC5835773; doi:10.1038/cr.2018.14)
Supplement: Supplementary information, Figure S10 — The cryo-EM density map of the splicing factors NY-CO-10 and RNF113A in the early, mature, and late Bact complexes [file cr201814x10.pdf]

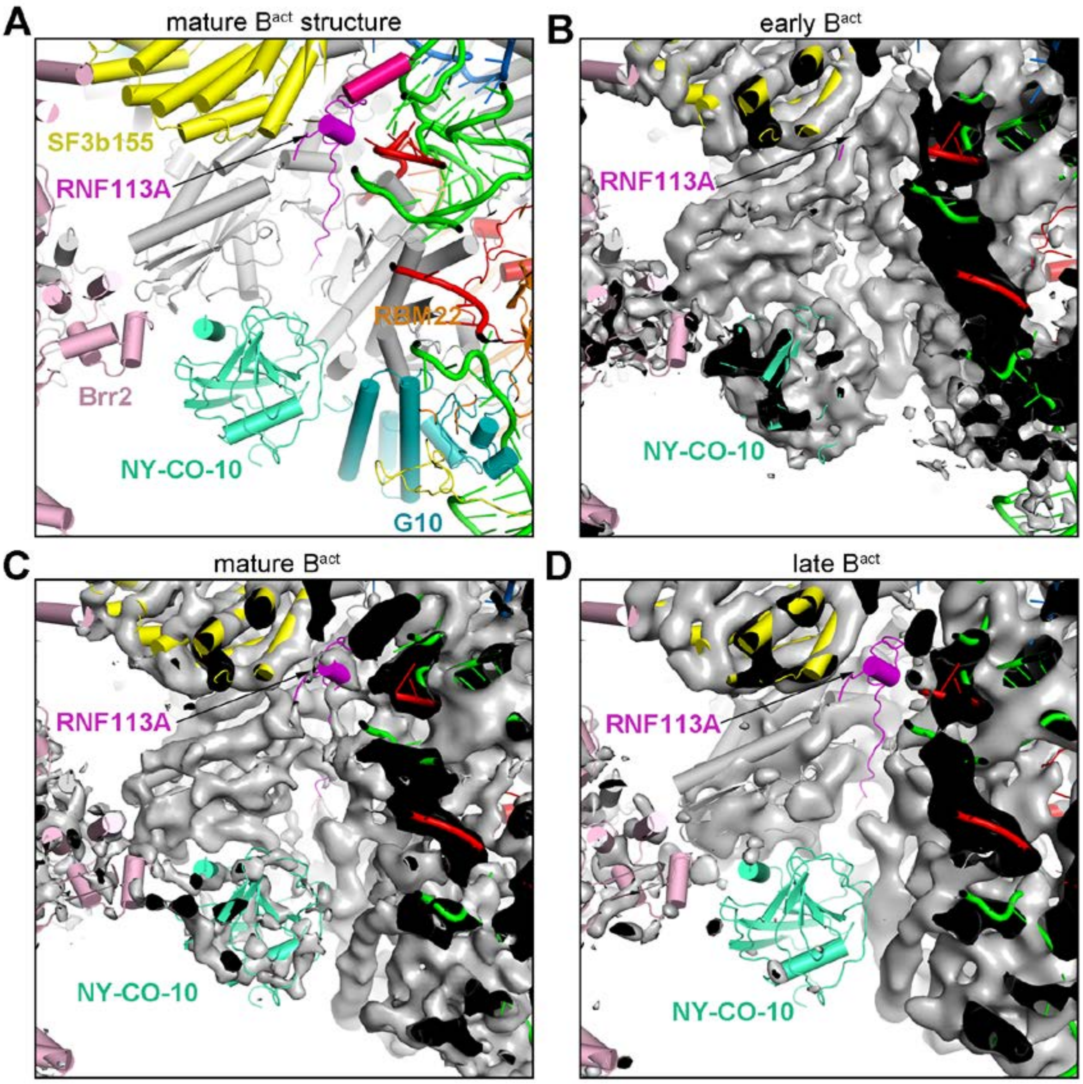

Figure S10 The cryo-EM density map of the splicing factors NY-CO-10 and RNF113A in the early, mature, and late B^{act} complexes. (**A**) The structure of the mature B^{act} complex around NY-CO-10 and RNF113A. NY-CO-10 (green cyan) and RNF113A (purple) are highlighted here. (**B**) A section of the 4.9-Å resolution EM density map of the early B^{act} complex around NY-CO-10 and RNF113A. There is strong density for these two proteins. The dinucleotide GU of the 5'SS is well protected by the N-terminal zinc-binding domain of RNF113A. (**C**) A section of the 5.1-Å resolution EM density map of the mature B^{act} complex around NY-CO-10 and RNF113A. The density for these two proteins is present but is no longer as strong as that in the early B^{act} complex. (**D**) A section of the 6.5-Å resolution EM density map

of the late B^{act} complex around NY-CO-10 and RNF113A. The density for these two proteins has completely disappeared, indicating these two proteins may have been dissociated.
