# Supplementary material for: Structure of the human activated spliceosome in three conformational states
Source: Cell Res. 2018 Jan 23;28(3):307–22. doi: 10.1038/cr.2018.14 (PMC5835773; doi:10.1038/cr.2018.14)
Supplement: Supplementary information, Figure S11 — The cryo-EM density map of the SF3a complex in the mature Bact complex [file cr201814x11.pdf]

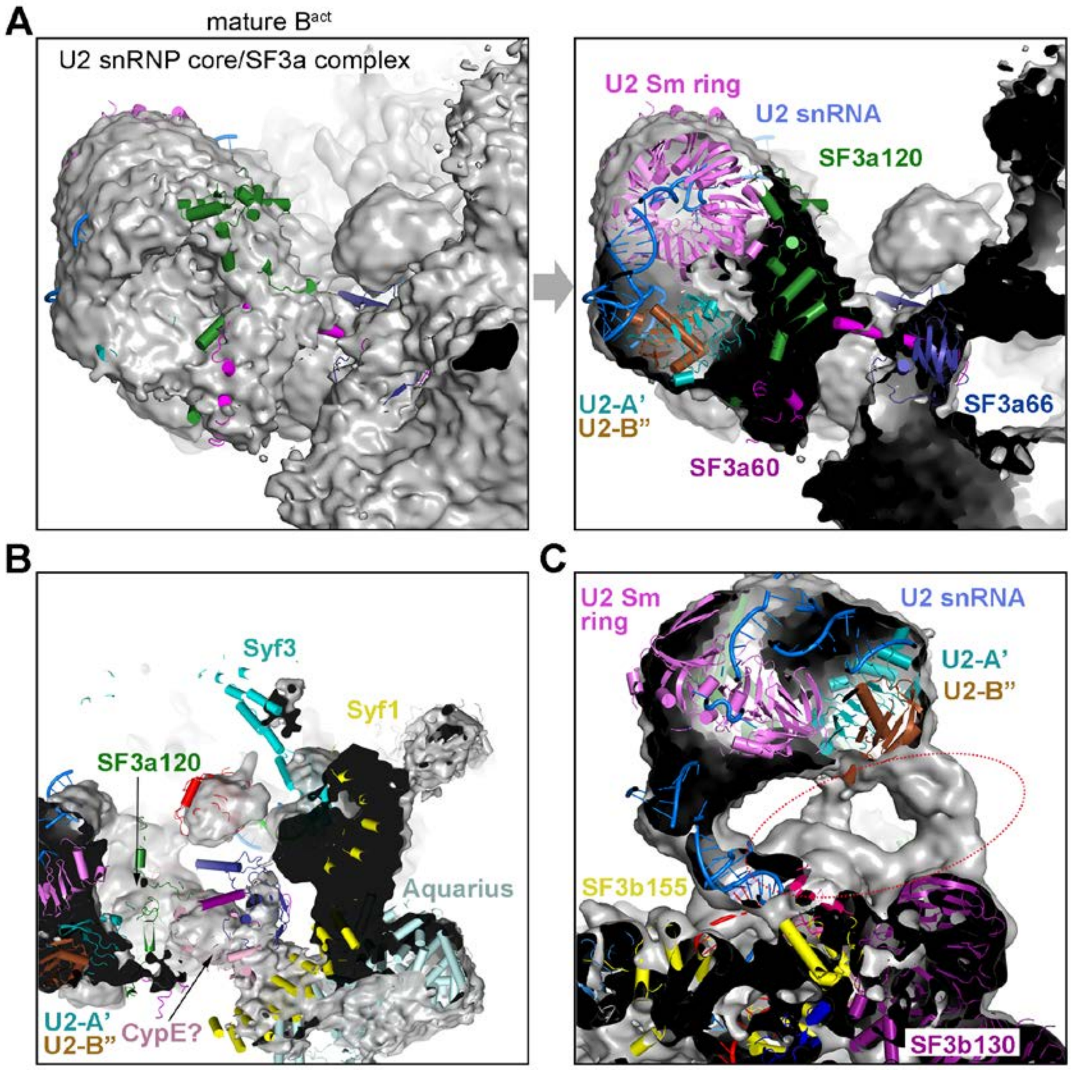

Figure S11 The cryo-EM density map of the SF3a complex in the mature B^{act} complex. (**A**) A close-up view of the EM density map on the U2 snRNP core and the SF3a complex. The extra density that is connected to the U2 snRNP core is likely derived from the unassigned region of the SF3a complex (left panel), and a sliced section of this region is shown (right panel). (**B**) A close-up view of the density map between U2 snRNP and Syf1/Aquarius. CypE appears to bridge the interactions between the U2 snRNP components and Syf1. (**C**) A close-up view of the density map that remains to be identified between the U2 snRNP core and the SF3b complex. These unknown proteins (within the dashed circle) bridge the interactions between SF3b130 and the U2 snRNP core.
