# Supplementary material for: Structure of the human activated spliceosome in three conformational states
Source: Cell Res. 2018 Jan 23;28(3):307–22. doi: 10.1038/cr.2018.14 (PMC5835773; doi:10.1038/cr.2018.14)
Supplement: Supplementary information, Figure S12 — Structure of SF3b155 in the Bact complex [file cr201814x12.pdf]

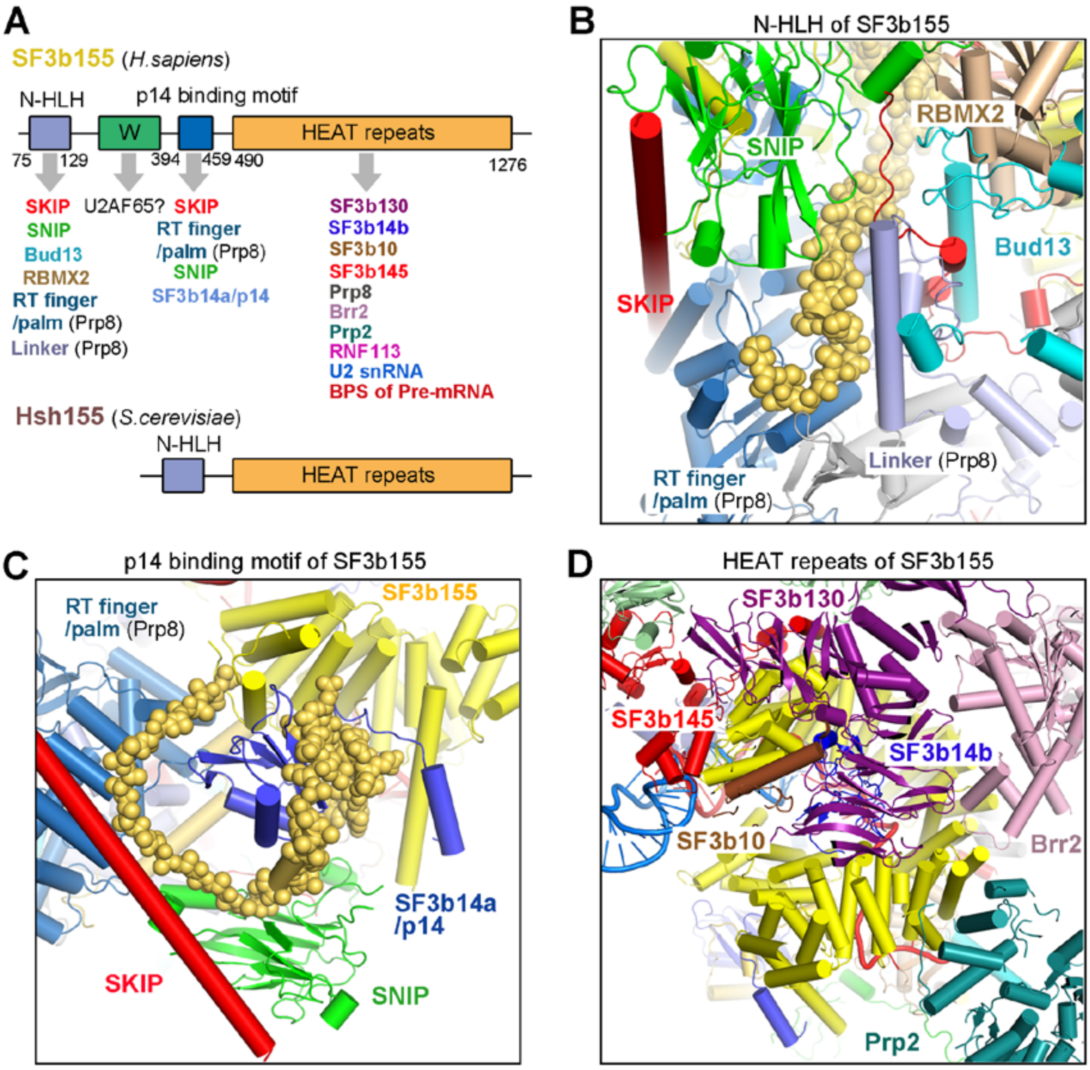

Figure S12 Structure of SF3b155 in the B^{act} complex. (**A**) The domain structure of SF3b155 and its interactions with the surrounding proteins. SF3b155 contains a helix-loop-helix (HLH) motif at the N-terminus, a Trp-rich region, a SF3b14a/p14-binding motif, and multiple HEAT repeats that form a superhelical domain. The functional orthologue of Hsh155 in *S. cerevisiae* lacks the Trp-rich region and the p14-binding motif. Accordingly, there is no functional orthologue of SF3b14a/p14 in *S. cerevisiae*. (**B**) A close-up view on the N-terminal HLH of SF3b155 and its interactions with surrounding components. The HLH is positioned between the RT Finger/Palm (marine) and the Linker (slate) domains of Prp8, and interacts with the RES complex (SNIP, RBMX2 and Bud13, colored by green, wheat and cyan,

respectively). (**C**) A close-up view on the p14-binding motif of SF3b155 and its interactions with surrounding components. This loop interacts with RT-Finger/Palm domain of Prp8, SKIP (red) and SNIP. (**D**) A close-up view on the HEAT repeat domain of SF3b155 and its interactions with surrounding components. In addition to binding the components of the SF3b complex, it also interacts with Prp2 (teal), Brr2 (light pink), and RNF113A (not shown here).
