# Supplementary material for: Structure of the human activated spliceosome in three conformational states
Source: Cell Res. 2018 Jan 23;28(3):307–22. doi: 10.1038/cr.2018.14 (PMC5835773; doi:10.1038/cr.2018.14)
Supplement: Supplementary information, Figure S13 — Comparison of the RNA elements among the B complex, the Bact complex, and the C complex [file cr201814x13.pdf]

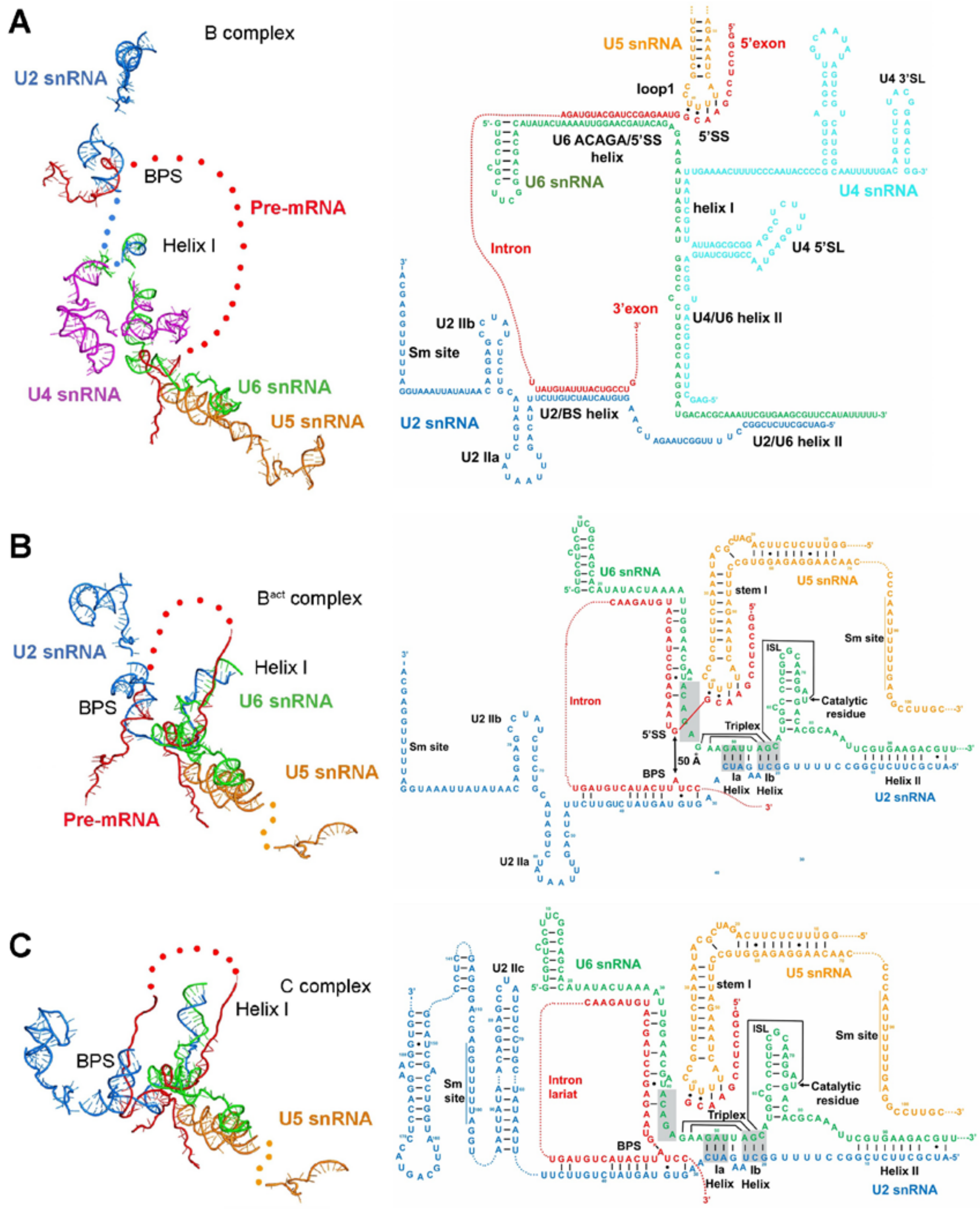

Figure S13 Comparison of the RNA elements among the B complex, the B^{act} complex, and the C complex. (**A**) Overall structure of RNA elements in the human B complex [21] (left panel) and the base-pairing specifics of the RNA map (right panel). Disordered RNA sequences are represented by red, dotted lines. (**B**) Overall structure of RNA elements in the human B^{act} complex (left panel) and the base-pairing specifics of the RNA map (right panel). (**C**) Overall structure of RNA

elements in the human C complex [24] (left panel) and the base-pairing specifics of the RNA map (right panel).
