# Supplementary material for: Structure of the human activated spliceosome in three conformational states
Source: Cell Res. 2018 Jan 23;28(3):307–22. doi: 10.1038/cr.2018.14 (PMC5835773; doi:10.1038/cr.2018.14)
Supplement: Supplementary information, Table S1 — Cryo-EM data collection and refinement statistics. [file cr201814x14.pdf]

Table S1. Cryo-EM data collection and refinement statistics.

**Data collection**	
EM equipment	FEI Titan Krios
Voltage (kV)	300
Detector	Gatan K2
Pixel size (Å)	1.338
Electron dose (e-/Å^2)	45.6
Defocus range (μm)	1.0~2.0
**Reconstruction (early/mature/late)**	
Software	RELION 2.0
Number of used Particles	96,523/27,405/14,316
Accuracy of rotation (°)	1.053/0.942/1.094
Accuracy of translation (pixels)	0.633/0.552/0.693
Final Resolution (Å)	4.9/5.1/6.5
**Model building**	
Software	Coot
**Refinement (early/mature,** against 4.9 Å and 4.8 Å map**)**	
Software	Refmac5.8
Map sharpening B-factor (Å^2)	-197.7/-179.4
Average Fourier shell correlation	0.900/0.900
R-factor	0.279/0.286
**Model composition (early/mature/late)**	
Protein residues	11,871/15,479/14,920
RNA nucleotides	414/414/414/
GTP	1/1/1/
**Validation (early/mature/late)**	
R.m.s deviations	
Bonds length (Å)	0.017/0.017/0.017
Bonds Angle (°)	1.792/1.803/1.802
Ramachandran plot statistics (%)	
Preferred	90.08/90.4/90.32
Allowed	6.72/6.63/6.71
Outlier	3.20/2.98/2.97
Clash score	40.2/40.1/40.1
Molprobity score	3.31/3.26/3.27
