# Supplementary material for: Structure of the human activated spliceosome in three conformational states
Source: Cell Res. 2018 Jan 23;28(3):307–22. doi: 10.1038/cr.2018.14 (PMC5835773; doi:10.1038/cr.2018.14)
Supplement: Supplementary information, Table S2 — Summary of model building for the human spliceosomal Bact complex. [file cr201814x15.pdf]

Table S2. Summary of model building for the human spliceosomal B^{act} complex.

	Molecule Human/S.pombe/S.cere	State early	mature	late	Length	Domain/Region	PDB code	Modeling	Resolution (Å)	Chain ID
**U5 snRNP**	U5 snRNA	√	√	√	117	8:71/85:104	5XJC	RD	4.0~5.0	B
	Prp8/Spp42/Prp8	√	√	√	2335	25:2335		RD	4.0~5.0	A
	Snu114/Cwf10/Snu114	√	√	√	972	54:943		RD	4.0~5.0	C
	Brr2	√	√	√	2136	404:2125		RD	4.0~9.0	D
	U5-40K/Cwf17/-	√	√	√	357	WD40 domain		RD	4.0~6.0	E
	SmB,D1,D2,D3,E,F,G	√	√	√	-	Sm fold		RD	6.0~15.0	a-g
**U6 snRNP**	U6 snRNA	√	√	√	107 nt	1:97	5XJC	RD	3.8~4.5	F
**Pre-mRNA**	Pre-mRNA	√	√	√	-	59 nt	5XJC	RD	4.0~6.0	G
**U2 snRNP**	U2 RNA	√	√	√	188 nt	1:47/54:184	5XJC	RD	4.0~20.0	H
	U2-A'/Lea1/Lea1	√	√	√	255	LRR domain	1A9N	RD	8.0~20.0	o
	U2-B"/Msl1/Msl1	√	√	√	225	RRM domain	1A9N	RD	8.0~20.0	p
	SmB,D1,D2,D3,E,F,G	√	√	√	-	Sm fold	4WZJ	RD	8.0~20.0	h-n
	SF3a120/Sap114/Prp21	√	√	√	793	SURP1/2,-	4DGW	RD	20.0~30.0	u
	SF3a66/Sap62/Prp11	√	√	√	464	ZF,-		RD	20.0~30.0	v
	SF3a60/Sap61/Prp9	√	√	√	501	-		RD	20.0~30.0	w
	SF3b155/Sap155/Hsh155	√	√	√	1307	HEAT repeat	5IFE	RD	3.8~4.5	1
	SF3b145/Sap145/Cus1	√	√	√	895	-	5GM6	HM	3.8~4.5	2
	SF3b130/Sap130/Rse1	√	√	√	1217	WD40 domain I/II/III	5IFE	RD	3.8~4.5	3
	SF3b49/Sap49/Hsh49	√	√	√	424	RRM domain I/II	5LSB	RD	6.0~15.0	4
	SF3b14a/p14-like/-	√	√	√	125	RRM domain	5IFE	RD	3.8~4.5	5
	SF3b14b/Ini1/Rds3	√	√	√	110	PHF5 domain	5IFE	RD	3.8~4.5	6
	SF3b10/SF3b10/Ysf3	√	√	√	86	-	5IFE	RD	3.8~4.5	7
**NTC/ Prp19 Complex**	Prp19	X	√	√	504	3:137	5XJC	RD	6.0~15.0	q-t
	Syf1/Cwf3/Syf1	X	√	√	855	TPR repeat		RD	6.0~15.0	I
	Syf3/Cwf4/Clf1	√	√	√	848	TPR repeat		RD	5.0~15.0	J
	Spf27/Cwf7/Snt309	X	√	√	225	14:222		RD	6.0~15.0	K
	Cdc5/Cdc5/Cef1	√	√	√	802	Myb domain/-	5XJC	RD	4.0~15.0	L
**NTC Related proteins**	G10/Cwf14/Bud31	X	√	√	144	1:144	5XJC	RD	4.0~6.0	N
	RBM22/Cwf5/Cwf2 /Ecm2/Cwc2	X	√	√	420	19:303		RD	4.0~6.0	O
	Ad002/Cwf15/Cwc15	√	√	√	229	2:78/188:229		RD	4.0~6.0	P
	Skip/Prp45/Prp45	√	√	√	536	51:316		RD	4.0~6.0	R
	Prl1/Prp5/Prp46	√	√	√	514	WD40 domain		RD	4.0~6.0	T
**IBC protein**	Aquarius/Cwf11/-	X	√	√	1485	19:1381	5XJC	RD	7.0~15.0	Q
**Known Splicing Factors**	Prp2	√	√	√	1061	ATP-dependent RNA helicase	5GM6	RD	8.0~15.0	S
	RNF113A/Cwf24/Cwc24	√	√	X	343	ZF	5XJC	HM	4.0~6.0	M
	SRm300/Cwf21/Cwc21	X	√	√	2296	1:26		RD	4.0~5.0	U
	Cwc22/Cwf22/Cwc22	√	√	√	908	MIF4G/ MA3		RD	4.0~10.0	V
**Step II factors**	Prp17	X	√	√	579	NTD (83:273);WD40 (274:576)	5XJC	RD	5.0~7.0	W
**RES complex**	SNIP1/-/Pml1	√	√	√	396	FHA domain	5GM6	RD	4.0~6.0	X
	RBMX2/Cwf29/Snu17	√	√	√	322	RRM domain		RD	4.0~6.0	Y
	Bud13/Cwf26/Bud13	√	√	√	619	-		RD	4.0~8.0	Z
**PPI proteins**	PPIL1/Cyp1/-	√	√	√	166	PPI domain	5XJC	RD	4.5~8.0	x
	CypE/-/-	X	√	√	301	RRM/PPI domain	3MDF/2R99	RD	8.0~15.0	y
	NY-CO-10/Cyp7/Cwc27	√	√	X	472	PPI domain	4R3E	RD	5.0~8.0	z

Under the column labeled "Molecule", proteins from human, *S. pombe*, and *S. cerevisiae* are colored black, red, and green, respectively. If the proteins from all three species have the same name, only a single name in black is indicated. Under the column labeled "Modeling", HM stands for homology modelling; RD stands for rigid docking.
